# Supplementary material for: Collective moderation of hate, toxicity, and extremity in online discussions
Source: PNAS Nexus. 2025 Nov 17;4(11):pgaf369. doi: 10.1093/pnasnexus/pgaf369 (PMC12659729; doi:10.1093/pnasnexus/pgaf369)
Supplement: pgaf369_Supplementary_Data [file pgaf369_supplementary_data.pdf]

Supplementary Information: Collective moderation of hate, toxicity, and extremity in online discussions

Jana Lasser & Alina Herderich, Joshua Garland, Segun Taofeek Aroyehun, David Garcia and Mirta Galesic

Disclosure: The supplemental appendix includes offensive language, which neither NAS nor the authors condone. Language is included as it appeared on the original social media posts used in the research project. If you do not wish to be exposed to offensive language as contained in the selected exemplary tweets, please skip pages 21 to 27.

Contents

S1 Related measures of discourse quality **3**
- S1.1 Toxicity . . . 3
- S1.2 Extremity of discourse . . . 3

S2 Classification scheme **5**
- S2.1 Definition of classification scheme . . . 5
- S2.2 Data-driven development . . . 6

S3 Labeling of training data **8**
- S3.1 Annotators . . . 8
- S3.2 Test set . . . 9
- S3.3 Training set . . . 9
- S3.4 Labeling process . . . 10
- S3.5 Labeling instructions . . . 12
- S3.6 Example tweets . . . 21
- S3.7 Interrater reliability . . . 28
- S3.8 Merging classes . . . 31

S4 Training the machine learning model **32**
- S4.1 Masked language modeling . . . 32
- S4.2 Fine-tuning and data augmentation . . . 32

S4.3 Training data overview 35

S5 Validation details 37
S5.1 Classifier performance on individual classes 37
S5.2 Determining classification thresholds for matching analysis . . . 40

S6 Descriptive statistics on the data set 42

S7 Trends in dimensions and quality of discourse over time 43
S7.1 Quality of discourse over time 43
S7.2 Argumentation strategies over time 45
S7.3 Ingroup/Outgroup content over time 46
S7.4 Emotional tone over time 47

S8 Relationship between discourse dimensions and quality 49

S9 Details of statistical analyses 55
S9.1 Micro level: Causal inference at the level of individual reply pairs 55
S9.2 Meso- and macro levels: Autoregressive distributed lag models . 56

S10 Relationship of discourse dimensions with quality of discourse 61
S10.1 Micro level 61
S10.2 Meso level 62
S10.3 Macro level 63

S11 Robustness analyses 64
S10.1 Analysis with longer reply trees 64
S10.2 Analysis stratified by user groups 67
S10.3 Robustness analyses overview 67

S12 Regression coefficients 71
S12.1 Tree-level ARDL 71
S12.2 Day-level ARDL 72

S13 REFORMS checklist 73

S14 Material Transfer Agreement 81

S1 Related measures of discourse quality

S1.1 Toxicity

To ensure that our conclusions do not depend on just one measure of discourse quality, we derived several related measures. One is a more general *toxicity* score, defined by Jigsaw's Perspective project as "a rude, disrespectful, or unreasonable comment that is likely to make you leave a discussion" [1]. We use Google's Perspective API [1] to measure the toxicity of tweets. Perspective was created by Jigsaw and Google's Counter Abuse Technology team as a tool to combat online toxicity and harassment [1]. The machine learning models that underpin Perspective were trained to identify a variety of characteristics in a piece of text e.g., whether that text is toxic, insulting, threatening, contains insults, or identity attacks. The perspective API takes a comment, tweet, utterance, etc. and returns the probability of it being in one of these classes, e.g., toxic. A higher score means that a user is more likely to perceive that piece of text as toxic. To accomplish this, Perspective's creators trained multilingual BERT-based models on millions of comments from a variety of sources, e.g., comments from online forums like The New York Times. To train these models, comments were labeled by 3-10 crowdsourced raters. The raters labeled whether a piece of text contained a characteristic (e.g., toxicity). They then derived a final label for each comment based on the ratio of raters who labeled a comment as e.g., toxic. For example, Perspective labels a piece of text as 0.6 for toxicity, if 6 out of 10 raters labeled a comment as toxic. One advantage of Perspective is that it covers many languages, including German, which makes it suitable for our data set. While Perspective outputs a probability for many different attributes, we focus our attention on measures that are conceptually most closely related to hate speech: toxicity, severe toxicity, profanity, insult, and identity attack. In our data set, these measures were highly correlated (median $r = .85$, minimum .56, maximum .96) and loaded on one factor. We therefore use the average of these measures as the overall toxicity score of a tweet. For more information on Perspective's design, features and implementation see [1].

Of the tweets in the data set, we were unable to assign toxicity scores to 17,384 tweets. This is because the tweet either had no text (e.g., because it consisted solely of an image), or the tweet was in a language that was not supported by the Perspective API. These tweets were excluded from all analyses.

S1.2 Extremity of discourse

We also track the *extremity of discourse*. Extreme speech about any topic is certainly acceptable by itself and can be valuable for a collective. However, when extreme positions are expressed in a way that alienates those who disagree, more moderate and opposing views can be suppressed. This in turn might further amplify the extreme positions as they can start to outnumber the other

voices. In contrast, an ideal discourse in the Habermasian sense would provide a good representation of public opinion rather than being biased towards any side because of suppression of other voices [2]. We measured extremity of speech as similarity of discourse to self-labeled speech of the members of Reconquista Germanica vs. of Reconquista Internet; for details see [3]. Members of these groups were typically expressing diametrically opposing views about current political issues, in particular about allowing immigration and the treatment of migrants already in the country, but also about different politicians and political events, as well as about other economic and broader societal issues.

Here we use the classifier developed in [3, 4] to measure two aspects of the extremity of discourse: the extremity of speech (that is, of tweets themselves), and the extremity of speakers (the overall extremity of all tweets of each speaker). The majority of tweets and speakers in the discussions we analyzed were rather neutral, that is, they were equally similar to both groups. Discourse similar to Reconquista Germanica was present in around 25 to 30% of tweets during the studied period, while discourse similar to Reconquista Internet was found in 13 to 22% of tweets (see [3] for details).

In [4], an ensemble classification system was developed to identify speech resembling the discourse of Reconquista Germanica (RG) and Reconquista Internet (RI) based on a data set of tweets from self-labeled RG or RI accounts. This classification system achieved accuracy scores in line with state-of-the-art results, on balanced test sets, and agreed with human judgment [3, 4]. We note that this classifier assesses the similarity of the text of a tweet to tweets that were posted by known RG or RI members and *not* the membership of the tweet author in either of these two groups. We use this classifier to approximate extreme speech reasoning that thought patterns are supposed to reflect in language, that is not only on the content level, but also on the level of speech characteristics [5]. For example, research using US congressional speeches has shown that Democrats and Republicans use different language to convey their positions [6].

The data used to train this classifier was different from the data set described in Section "Data set" in the Methods in the main text, although it stemmed from the same period. It included more than 9 million relevant tweets originating from timelines of known RG accounts (4,689,294 tweets) or RI accounts (4,323,881 tweets). In an earlier research project, this data allowed us to build an ensemble classifier in which each classifier consisted of a fine-tuned doc2vec model [7] coupled to a regularized logistic regression function. In total, 289 unique classifiers were trained, each of these used different hyperparameters and slightly different training data. The 25 top-performing classifiers were combined to form the final ensemble classification system by averaging, for each tweet, their estimates of the probability that the tweet resembles RG's or RI's discourse. For more details about the classification system's construction, training and accuracy see Ref. [4]. Here, we classify tweets that have an average probability of 0.7 or higher to resemble either RG's or RI's discourse as "extreme speech".

S2 Classification scheme

The first step in the development of the machine learning classifiers was to develop a classification scheme that defines and characterizes the variables we aimed to detect. In the classification scheme, HATE (as a measure for the quality of discourse) signifies whether a tweet includes hate speech. STRATEGY (dimension of discourse) encodes the argumentation strategy employed in a tweet. In- and outgroup thinking is represented by GROUP, which reflects whether a comment addresses the in- and/or outgroup of a speaker. GOAL (dimension of discourse) reflects the socio-psychological goal of the speaker with respect to their in- and outgroup and is only defined if an in- or outgroup is addressed. Please note, that we only use the classifier based on GOAL to measure "in- and outgroup content" later in the process.

S2.1 Definition of classification scheme

Each of HATE, STRATEGY, GROUP, and GOAL contains several *classes*. For example, STRATEGY contains the classes “opinion”, “constructive”, “sarcasm”, “leaving factual discussion” and “other”. Consequently, for each of HATE, STRATEGY, GROUP, and GOAL we trained a separate machine learning classifier to detect these classes (see Section “Measurement of discourse quality and dimensions of discourse” in the Methods in the main text and Section S4 for details). In the context of machine learning and training a classifier to identify classes we will talk about “labels” that are given to individual tweets that assign a class to the tweet. A classified tweet has a label for HATE, STRATEGY, GROUP, and GOAL, respectively. Therefore, a tweet can for example be classified as containing hate speech (HATE), employing a certain argumentation strategy (STRATEGY), and mentioning an outgroup (GROUP) to achieve a socio-psychological goal (GOAL).

We developed the classification scheme *classifier-agnostic*, which means that the scheme emphasizes truthfulness and completeness of categories rather than focusing on the feasibility for machine learning training. Importantly, we wanted to reflect the abundance of online political discourse with the deliberate decision to have a fine-grained classification scheme with many classes and merge classes later in the process if necessary to train the machine learning classifiers. It is important to note during data labeling the annotators did not know the political orientation of the person who posted a tweet and were instructed to judge the presence of hate independently of the political slant they could detect in the tweet. We used these human ratings to train a classifier to predict the probability of hate speech in the rest of the tweets in our data set (see Section "Measurement of discourse quality and dimensions of discourse" in the Methods in the main text and Section S4 for details).

Where applicable, the classification scheme was derived theory-driven for HATE, GROUP, and GOAL. On the other hand, STRATEGY was developed in a data-driven way, following a grounded theory approach [8] as existent classifications from discourse analysis are not specific to counter speech [9] or

differ in their understanding of what counter speech is. For example, Benesch and colleagues [10] expect counter speech to shift the opinions of hateful users, an expectation we find unrealistic. Instead, we define counter speech as an attempt to counter concrete instances of hateful speech in online conversations with the goal to influence public norms towards a more civilized and fact-based discussion.

We added the class "uninterpretable" to each of HATE, STRATEGY, GROUP, and GOAL to account for tweets that could not be classified due to missing context. For example, in the case of GROUP, it was not always apparent which group the speaker identifies with, making it impossible to determine whether they were referring to an outgroup in their tweet. If multiple classes were worth considering, annotators were supposed to assign the dominant class, i.e., the class that they felt was most likely to be true. Accordingly, "uninterpretable" was only assigned if the annotators felt that two or more classes were equally likely.

If a tweet was classified as hate speech, annotators also labeled the targeted group, for example, left- or right-wing parties, institutions, or vulnerable groups such as immigrants. We understand this addition to the classification scheme as a precaution: Although we didn't end up using those labels in our analysis, the goal was to maintain fairness towards different political orientations while being able to discern potentially unequally detrimental forms of hate speech. In other words, while hate speech against German right-wing political groupings exists, hate speech against vulnerable groups such as immigrants will be more harmful due to the distribution of power within the society. Fig. S8 shows the relative distribution of targeted groups over time.

Regarding in- and outgroup thinking (GROUP), we did not constrain the definition to specific group qualities such as political orientation, but acknowledged that in- and outgroup thinking can be activated with respect to other characteristics such as ethnic identities, or on the level "government versus people".

GOAL was only labeled for tweets where GROUP is not labeled as "neutral" or "uninterpretable". Although GROUP and GOAL correspond to some extent, we did not enforce certain label combinations, e.g., GROUP = "out" often goes together with GOAL = "weak", although a tweet can very well address the ingroup (GROUP = "in"), while aiming at weakening the outgroup (GOAL = "weak"), too. We note that GROUP is only used to identify tweets for which GOAL is labeled and does not enter the statistical analyses described in Section "Statistical analyses" in the Methods in the main text.

S2.2 Data-driven development

The classification scheme was developed by three annotators under the leadership of AH, who was also one of the annotators. All of the annotators were native German speakers with broad political interest and were advanced master-level psychology students at the University of Graz, Austria or had a master's degree in psychology.

Based on manual inspection of a random sample of $n = 1,000$ tweets from the corpus, we developed the classes of STRATEGY. In particular, we focused on the question of which argumentative means a speaker used in order to influence public discourse in favor of their view. The initial sample of tweets was evenly distributed across time and extremity of speakers (see Section S1.2 for details). With this first version of a classification scheme, two annotators classified the initial sample of $n = 1,000$ tweets independent of each other. We then compared the assigned labels, agreed upon ambiguous tweets, and refined the classification scheme including merging or creating new classes until all annotators felt that the classification scheme was complete. This process was repeated until adequate interrater reliability was achieved with respect to the annotation task and expectations from previous studies (see [11] and [12]). Interrater reliability is reported and discussed in more detail in Section S3.7 below.

For HATE, GROUP and GOAL, we started with sets of theory-driven classes. All three annotators classified 10% of tweets (i.e., $n = 100$) from the initial sample for HATE, GROUP and GOAL in order to test the feasibility of the theory-driven class definitions. Similar to STRATEGY, all annotators discussed edge cases and refined classes as well as their in- and exclusion criteria as needed.

The final classification scheme including fine-grained classes alongside the coarser grained merged classes the machine learning classifiers were trained to classify (described in Section S4) is summarized in Tab. S1. Exemplary tweets for each class are provided in Section S3.6.

Table S1: **Overview of the classification scheme**. Final classes the machine learning classifiers were trained to classify (first column), sub-classes according to the classification scheme included in these classes (second column) and descriptions of the sub-classes (third column). The instructions for human annotators provided in Section S3.5 include detailed descriptions with inclusion and exclusion criteria, and example phrasings for all classes.

Merged class	Class	Class description
	**HATE** (quality of discourse)	
Taken together, would you say this tweet contains hateful speech?		
yes	hate speech	contains hateful speech according to the definition
no	no hate speech	does not contain hateful speech according to the definition
	uninterpretable	could be hateful depending on the context
	**STRATEGY** (dimension of discourse)	
Which argumentative means does the speaker use to influence public discourse in favor of his/her view?		
opinion	opinion	expressing a not necessarily objective opinion without insults
constructive	information	providing factual information which is verifiable or falsifiable
	question	asking a honest question or seek further information

	consequences	pointing out realistic or unrealistic negative consequences
	correcting somebody	calling somebody out for behavior or choice of words
	inconsistency	exposing hypocrisy or revealing contradictions
sarcasm	sarcasm	umbrella term for sarcasm, irony, cynicism
leave fact	personal insult	insulting a particular person with name-calling or profanities
	-isms insult	racism, sexism, antisemitism, homophobia; insulting a group based on innate characteristics
	political insult	insulting political figures using derogatory political terms
	institutional insult	portraying state, media or science as useless or corrupted
other	uninterpretable	the comment is ambiguous with respect to all other categories
	other	none of the above (e.g. genuine humor, popcultural references)
foreign	foreign language	comments not in German
	**GROUP** (dimension of discourse)	
Does the speaker address their ingroup or outgroup?		
out	outgroup	addressing the speaker's outgroup
not out	ingroup	addressing the speaker's ingroup
	both	addressing both in- and outgroup of the speaker in equal terms
	neutral	speech without signs of in-/outgroup thinking
	uninterpretable	speech with signs of in-/outgroup thinking, where the speaker's identity is not apparent
	**GOAL** (dimension of discourse)	
What is the socio-psychological goal of the tweet?		
exclusionary about outgroup	threat	pointing out realistic or unrealistic threats from the outgroup
	weak	making members of the outgroup look stupid
inclusionary about in/both groups	strengthen	highlighting positive characteristics of ingroup
	justify	justifying actions of ingroup
	common ground	pointing out common characteristics of in- and outgroup
	common problems	pointing out common challenges of in- and outgroup
other	not applicable	assigned if GROUP is labeled *neutral* or *uninterpretable*

S3 Labeling of training data

S3.1 Annotators

We started the labeling process with the same three annotators that were involved in the development of the classification scheme (see Section S2). However, after labeling about 50% of tweets necessary to create the training data set, one annotator was replaced by a fourth annotator with similar qualifications as the other annotators. The annotator was trained by labeling tweets

that were already labelled by the other annotators and discussing disagreements with AH. As soon as interrater reliability reached the levels of reliability established by the first three annotators, the new annotator took over regular labeling tasks. For a more in-depth discussion of interrater reliability see Section S3.7.

S3.2 Test set

To create a held-out test set for the final validation of the classifiers (see Section "Validation of machine learning classifiers" in the Methods in the main text and Section S5 for details), we drew another random sample of $n = 1,000$ tweets balanced across time and extremity of speakers. All four annotators independently labeled the test set and annotators were not allowed to discuss specific tweets in the test set. We report Krippendorff's alpha for the test set in Tab. S3.

To create the ground-truth test set for validation of the classifiers we only used labels on which at least three out of four annotators agreed. The final test set included 900 examples for hate speech and 677 examples for argumentation strategy. For in- and outgroup thinking, too few examples of the class "inclusionary about in/both groups" were included in the original held-out test set to allow for a reliable validation of the classifier for this class. To resolve this issue, we created a second test set for which we drew a random sample of 200 tweets from the unlabeled data that was biased towards the "inclusionary about in/both groups" class and asked two annotators to independently label the sample. Krippendorff's alpha between the two annotators on this data set was in line with the interrater reliability values observed for the other labeling tasks (see Section S3.7 below for details). To create the test set for GOAL, we used examples where both human raters agreed (127 examples, out of which 23 were "inclusionary about in/both groups", 44 were "exclusionary about outgroup" and 60 were "neutral/unint").

S3.3 Training set

Throughout the labeling process we took an iterative approach to sample each new batch to be labeled. Specifically, we used a preliminary text classification algorithm (a support vector machine trained on term-frequency-inverse-document-frequency embeddings of all available labeled data) to tailor the sampling of data for human annotation such that the balance of class frequencies in the training data was improved. This approach was necessary since some classes (for example the class "constructive" of STRATEGY) were very rare. We describe the organization of the sampling and labeling process below.

As first step, the initial sample of 1,000 tweets that was used to develop the classification scheme (see Section S2) was re-labeled with the finalized classification scheme by two annotators. This labeled data set was used to train a preliminary classifier for STRATEGY. The trained preliminary classifier was used to infer labels for STRATEGY for the remaining unlabeled tweets. The

next batch of data to be labeled by the annotators was then composed by oversampling minority classes in STRATEGY based on the inferred labels. Newly labeled batches were again used to train the support vector machine to gradually improve its performance and hence the bias towards minority classes in subsequent batches. We progressed in batches of 500 tweets per annotator at a time.

We decided to bias the sampling towards minority classes of STRATEGY because it is most directly related to our research question to assess the effectiveness of different counter speech strategies. Furthermore, some of the minority classes in STRATEGY correlate with HATE (e.g., '-isms insult' is often used to express hate speech). In addition, GROUP and GOAL are less fine-grained and we expect in- and outgroup thinking to occur over different forms of argumentation strategies.

In general, each tweet was labeled by a single annotator. We justify this decision based on the fact that we worked with expert annotators, who were expected to produce annotations of higher quality than traditional crowd sourced annotators like workers on Amazon Mechanical Turk. Furthermore, adequate interrater reliability in the test batch suggests sufficient similarity of labels among raters. Restricting the labeling to one label per tweet allowed us to obtain more labeled data given the restricted budget for labeling, generating more diverse training data for the machine learning classifier that helped improve classifier generalizability. We use computational data augmentation approaches (see Section S4.2) to supplement labels by single human annotators with inferred labels from preliminary classifier versions to increase the size of the training data set.

To ensure that the conceptions of classes were not drifting apart between individual human annotators over the extended time it took to complete the labeling ($\sim$ 9 months), 10% of tweets from each batch ($n = 50$) of each annotator were labeled by a second annotator. We calculated Krippendorff's alpha on the tweets with two labels to track the interrater reliability over the course of the labeling process. We provide interrater reliability values for each labeled batch in Fig. S2.

S3.4 Labeling process

We followed an iterative process to select examples for human coding from the corpus with the aim of finding enough examples from rare classes. The sampling and labeling process is visualised in Fig. S1. We developed and refined the initial classification scheme in the "development batch", consisting of 1000 randomly drawn tweets. After the classification scheme was finalised, an additional batch of 1000 tweets was randomly drawn from the corpus and labeled by all human annotators. This batch serves as the held-out test set and is not used for training the machine learning classifiers. We then trained a first version of a machine learning classifier to classify tweets for STRATEGY based on the "development batch". We used labels inferred by this preliminary classifier to draw additional batches of examples (500 tweets per annotator) for human

annotation that were biased towards rare classes in STRATEGY. These tweets were only labeled by a single human annotator except for 50 tweets, which were labeled by a second annotator. This process of training a new classifier version on the labeled data and drawing a new batch of examples for human annotation was repeated 14 times. For each batch, the subset of tweets that was labeled by two annotators was used to monitor interrater agreement throughout the annotation process and to determine "confident examples" during classifier training (see Section S4.2). Figure S2 shows Krippendorff's alpha values for each of HATE, STRATEGY, GROUP, GOAL and TARGET (not used in the main analysis) throughout the labeling process (see also Section S3.7 below).

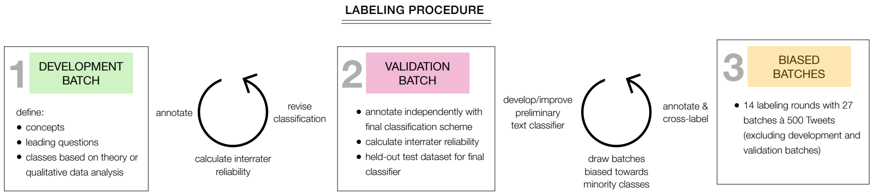

Fig. S1: **Schematic depiction of the labeling procedure.** The development batch is used to develop the classification scheme, which is revised in a circular process. It also serves as the basis for a preliminary text classifier that is trained to bias the data to be labeled towards minority classes. We biased towards minority classes in STRATEGY.

S3.5 Labeling instructions

1. [STRATEGY] Which argumentative means does the speaker use to influence public discourse in favor of his/her view?

Definition: As argumentative means we understand the combination of a particular content (what) expressed in a particular way (how).

ANNOTATION PRINCIPLES

1. Be very sensitive to racism (or other derogatory statements solely based on group membership). But still try to see the best in people.
2. Consider that verbal ability varies. Comments might not necessarily be rude, just because they are not very elevated.
3. Reflect on your own political position. Even if you disagree with a tweet, refrain from prejudging and really try to focus on the strategy the author is using.
4. Assign one label only. Even though some tweets might be a mix of strategies, try to assign a label based on the main focus of the tweet.

— CONSTRUCTIVE COMMENTS —

info
information

- Providing factual information. Other than expressing an opinion this content is verifiable or falsifiable.
- Label „misinformation agnostic“. Even though the provided fact might not be true, the author might have tried his/her best to contribute to a factual discussion.
- tweets in this category often contain links, but must not necessarily be supported by external resources, i.e. can just be formulated as what a person thinks he/she knows.

/ includes:
citations, giving definitions (e.g. of legal regulations)
/ involves implicit or explicit expressions such as:
„ Zu beachten ist, dass ...“, „Interessant ist auch ...“

opin
opinion

- Expressing an honest opinion without insults. The claim must not necessarily be objective or provide reasons.
- There are different types of opinions (see Kharde & Sonawane, 2016):

  1. opinion: a conclusion open to dispute
  2. view: subjective opinion
  3. belief: deliberate acceptance and intellectual assent

4. sentiment: opinion representing one's feelings

- Label opinion whenever you feel you could rephrase the message in the following way: „The way I see it is …“, „I think that ….“ or „I have the feeling that …“.
- The tweet may involve harsh or provoking language.

/ includes:
proposing solutions for problems, complaining about failures (e.g. of politicians), tweets seeming to be a response to someone else as long as the tweet can be understood as alone-standing opinion, disagreeing with someone, agreeing with someone, rhetorical questions that are used to make a point as long as they are not sarcastic, idioms & folk wisdom, emotional expressions (i.e. expressing negative or positive feelings), calls to action, acknowledging the media, journalists or expert opinions

quest
question

- Ask an honest question. Seek further information or clarification.
- Only assign that label if you expect the author to be open for actual responses.

/ includes:
asking someone else to elaborate further, questions one had that have already been answered
/ distinguish from:
rhetorical questions

conseq
consequences

- Pointing out (realistic or unrealistic) negative consequences of certain decisions, actions or circumstances.

/ includes:
comparisons with other similar circumstances
/ involves implicit or explicit expressions such as:
„Ihr werdet sehen, was passiert, wenn die alle ins Land kommen!“, „Wenn man den Rechten weiter erlaubt durch die Städte zu marschieren, dann …“

correct
correcting somebody

- Calling somebody specific out for his or her behavior or offensive language, or calling somebody out for a specific instance of misbehavior.
- Includes everything involving choice of words.

/ includes:

opposing insults, telling people minding their language, correcting somebody for spreading misleading content or misinformation, reminding people of the actual meaning of their tweet
/ distinguish from:
disagreeing with an opinion > label opin

inconsist
inconsistency

- Stating that appearance and behavior/agenda of someone contradict each other.
- Exposing hypocrisy.
- Keep in mind: The intention of the speaker to point out inconsistencies/hypocrisy counts, not if the described situation actually is inconsistent.

/ includes:
telling someone that it is not his or her place to make a certain statement, exposing untruthful PR language, whataboutism (this means the points that the speaker is comparing do not necessarily have to be related)
/ involves implicit or explicit expressions such as:
„Wenn man es Systemkritik nennt, wird verharmlost, was die AfD wirklich ist!“, „Sie sagen, Sie halten sich an die Regeln, aber auch Abschiebungen können legal sein!“

— LEAVING FACTUAL DISCUSSION —

sarc
sarcasm

- Sarcasm is used as an umbrella term and includes sarcasm, irony and cynicism concerning a particular topic or circumstance.
- (Attempt of a definition) Sarcasm means that you say something that you actually don't mean in order to point out the exact opposite. Can have a touch of hopelessness.
- (Attempt of a definition) Cynicism is a comment formulated in a mocking or provoking fashion. Content might intentionally violate societal norms.
- (Attempt of a definition) Irony is a more general term of people saying something that they don't mean.
- Rhetorical questions often have a sarcastic notion, however they can be classified as opinion, too.
- Rule of thumb (1): Consider the content of the tweet: How „unrealistic“ is it? (e.g. „Wenn die Welt sowieso den Bach runter geht, können wir uns auch gleich alle gegenseitig abknallen.“ > pretty sarcastic; „Naja, was soll die alleinerziehende Mutter bei solchen Gesetzen auch machen?“ > points out the financial struggles of single parent mums and is therefore less sarcastic and could be labeled opin)

- Rule of thumb (2): If you are unsure whether a tweet is opin or sarc, ask yourself: How sarcastic is this tweet on a scale from 0 to 10? > if 5 or greater, label sarc

/ distinguish from:

- being sarcastic, ironic or cynic against a person > label insult-pers
- if a rhetorical question is used to convey an opinion > label opin

insult-pers
personal insult

- Insulting a particular person with name-calling (i.e. abusive words or derogatory statements) or profanities (i.e. obscene or rude language).

/ includes:
deny a politician's competencies in general; sarcastic, ironic or cynic comments about a person or group of people, ridicule somebody, threatening somebody
/ distinguish from:
if insult refers to a political group or orientation > label insult-polit

insult-ism
-isms insult

- Statements including racism, sexism, antisemitism, homophobia or statements insulting an entire group of people solely based on group membership (innate characteristics).

/ distinguish from:
if the statement insults entire political parties or groups > label insult-polit

insult-polit
political insult

- Insulting political opponents using derogatory political terms. Can refer to individuals or groups.
- Generalizing statements that can be subsumed under „All leftist people are ...“, „All rightist people are ...“.

/ includes:
referring to an entire political group as unintelligent in any way, claiming a politician to be incompetent
/ involves implicit or explicit expressions such as:
„linksgrünversifft“, „braunes Gesindel“
/ distinguish from:

- if the tweet is claiming a politician to be incompetent in general > label insult-pers
- if insults are used > political insults: label insult-polit; other insults: label insult-pers

- if media is accused of suffering from left or right-wing bias > label insult-inst

insult-inst
institutional insult

- Insulting public or political institutions, the state or the media, as well as science or scientists as a whole.
- Whenever a statement refers to „all politicians“, „all media“, etc.
- Only refers to the system that the person lives in (i.e. Germany, Europe).
- If foreign institutions are insulted, do the following checks:
- (1) Is it insult-ism? If the institutional insult is based on racist attitudes. Example: „Von Regierung XY braucht man sowieso nichts erwarten, in Afrika sind ja eh alle korrupt.“
- (2) Is it insult-pers? If a head of state is insulted with profanities. Example: „Erdogan ist ein beschissener Diktator mit Spatzenhirn.“
- (3) Is it opin? If a realistic threat is pointed out in a rather neutral fashion. Example: „Erdogan ist ein Diktator, der die Meinungsfreiheit unterdrückt. So einfach ist das.“
- (4) If it doesn't fit under (1) to (3), still label opin.

/ includes:
calling them incompetent, questioning their right of existence, implying their uselessness, questioning the independence of the media, defaming scientists

— OTHER —

other
other
/ includes:

- The annotator doesn't understand the meaning of the tweet in the sense that the tweet is „gibberish“.
- genuine humor
- technical posts
- tweets about daily life, travel
- pop cultural references or tweets reacting to Neo Magazin Royale content

unint
uninterpretable

- The tweet is ambiguous with respect to the other categories, i.e. hard to interpret without further context.
- Before assigning the label try your best to decide for another one.

/ includes:
incomplete tweets (e.g. tweets within threads), many @janboehm related tweets as it is unclear if it is a humorous or an insulting tweet

Remark:

tweets in a foreign language (i.e. not German) are assigned the label foreign

2. [GROUP] Does the speaker address his/her ingroup or outgroup?

outgroup / ingroup	yes	no
yes	both	out
no	in	neutral

General rule:
Try to avoid the label neutral. As long as you have the feeling that in/out-group thinking is activated in any way, do not label neutral.

in
ingroup
addressing the speaker's own group (many group-defining characteristics are possible, e.g. political orientation)
/ includes:
tweets about individuals representing a certain group, e.g. the head of a party being addressed instead of the party as a whole

out
outgroup
addressing a group the speaker does not belong to (many group-defining characteristics are possible, e.g. an opposing political orientation)
/ includes:
tweets about individuals representing a certain group, e.g. the head of a party being addressed instead of the party as a whole

both
both (in- and outgroup)
addressing both an in- and outgroup of the speaker in the same tweet
/ includes:
tweets comparing in- and outgroup directly; if the speaker reveals multiple of his/her group memberships

neutral
neutral
speech addressing neither in- nor outgroup, for example non-political comments

unint
uninterpretable
the tweet addresses a certain group, but which group the speaker belongs to is not apparent without further context

3. [GOAL] What is the social psychological goal of the tweet?

strengthen
strengthen ingroup
putting own group in a positive light; portraying own group as superior
/ includes:
intimidation; posturing; giving own group appealing labels; bragging about own group; clarifying the image of own group
/ involves implicit or explicit expressions such as:
„There are many of us.“; „We are the last defense of our nation.“; „We are patriots.“; „We are the smart ones.“

justify
justifying actions of ingroup
justifying actions of own group
/ includes:
dehumanization of other group; stating that own behavior was a reaction to the outgroup's; depriving the outgroup of their rights
/ involves implicit or explicit expressions such as:
„We have to defend ourselves.“; „We need to put ourselves first.“

threat
highlight a threat from outgroup
pointing out realistic or unrealistic threats of outgroup
/ includes:
concrete, as well as symbolic threats; giving other group threatening labels; warning of dictators or non-democratic systems
/ involves implicit or explicit expressions such as:
„They are taking our jobs.“; „They are attacking our women.“; „German values are vanishing.“; „Islam is taking over.“

weak
make outgroup look weaker
making members of other group look stupid, irrational, backwards
/ includes:
pointing out failures of the other group

common ground
emphasize common ground
pointing out overlapping characteristics of in- and outgroup to make a point
/ involves implicit or explicit expressions such as:
„I am also German, but I value immigrants.“; „I am also an immigrant, but I disagree with accepting more immigrants.“

common problems

emphasize common problems
pointing out challenges that in- and outgroup face as a community
/ includes:
asking the outgroup for their honest opinion, making a discourse effort, if ingroup criticizes itself
/ involves implicit or explicit expressions such as: „We should actually be talking about climate change.“

Remark:
If GROUP is labeled as neutral or unint, GOAL doesn't have to be assigned.

4. [HATE] Taken together, would you say this tweet contains hateful speech?
[TARGET] If yes, please specify the group that the tweet is hateful against.

Hate speech definition:
Hateful speech includes insults, discrimination, or intimidation of individuals or groups on the Internet, on the grounds of their supposed race, ethnic origin, gender, religion, or political beliefs. It also includes speech that aims to spread fearful, negative, and harmful stereotypes, call for exclusion or segregation, incite hatred, and encourage violence against a particular group.

General rule of thumb:
Does this tweet „feel hateful“? > If yes, check the definition to see whether your initial feeling can be justified by any of the criteria.

Groups:
institutions // **inst**

- including state, media & science

political parties
right wing parties (generally speaking: FDP, CDU, CSU, AfD) // **right wing**
left wing parties (generally speaking: SPD, die Grünen, die Linke) // **left wing**

- when labeling, consider the standpoint of the speaker, e.g. from an AfD supporter's perspective, Angela Merkel will be left wing versus from a Green Party supporter she may be right wing
- personal insults of politicians or public individuals count as hate speech if they are addressed as a representative of their group (depends on the phrasing of the tweet)

ethnicity and race
privileged ethnic groups (in the cultural context of Germany) // **privileged**
vulnerable ethnic groups (in the cultural context of Germany) // **vulnerable**

religion
religious majority (in the cultural context of Germany, i.e. Christian) // **rel-maj**
religious minority (in the cultural context of Germany, i.e. Muslims, Jews, other) // **rel-min**

gender // **gender**

age // **age**

Remark:

- if it is not clear, if the tweet is hateful because of missing context > label unint
- if the tweet is hateful, but against which group is unclear > label [TARGET] unint

S3.6 Example tweets

Table S2: **Exemplary tweets for all subcategories in the classification scheme**. All examples contain hate speech. German originals are provided with an English translation. The spelling of the original tweets is left unchanged. HATE and TARGET are listed together as TARGET is a specification of HATE. We also present GROUP and GOAL in combination as some labels frequently co-occur.

German original	English translation
**HATE & TARGET (Is the tweet hateful and if yes, against which group?)**	
institution	
@tagesschau Dreckslügenpresse Hochverräter fahrt zur Hölle	@tagesschau Filthy Lying Media high traitors go to hell
right wing parties	
Hauen Sie ab #Petry u. #Weidel u. #AfD statt mit Euren grosskotzigen Mündern zu belästigen oder 1 andere Nation. Pseudos- #DefendEurope	Fuck off #Petry a. #Weidel a. #AfD instead of molesting with your swanky mouths or another nation. Pseudos- #DefendEurope (note: Alternative für Deutschland, Alternative for Germany, German right-wing party; Petry and Weidel are/were two high-ranking members of the party)
left wing parties	
Die Grünen sind überflüssig wie Fußpilz.	The Green Party is as unnecessary as athlete's foot.
vulnerable ethnic groups	
Die Politik sollte sich besser darauf konzentrieren diese Kulturbereicherer alle wieder loszuwerden	Politics should rather concentrate on getting rid of all those culture enriching people again
religious minority	
@vinterblot1985 Muslime sind abfällig gegen Juden +aggressiv+gewalttätig. Der Antisemitismus ist da ausgeprägter. Fragen sie Juden selbst	@vinterblot1985 Muslims are derogatory against Jews +aggressive+violent. Their anti-Semitism is more pronounced. Ask the Jews themselves
privileged ethnic groups	
Apropo Ziegen Ficker Kultur stammt aus Deutschland.	By the way goat fucker culture comes from Germany.

religious majority	
Kann dem Kinderschänderverein nix abgewinnen.Die sollen sich aus der Politik raushalten das ist Gesetz hier !	Cannot get anything out of this child abuser club.They should keep out of politics this is the law here !
gender	
Frauen können keine politik....siehe Merkel	Women aren't capable of doing politics....see Merkel
age	
Lernt die Jugen lieber mal Sätze zu sprechen und Kopfrechnen	Learn the youth better to articulate sentences and mental arithmetic
uninterpretable	
Sehen Sie sich die Türkei an. Das ist der Islam. Sehen Sie sich alle mehrheitsmuslimischen Länder an. Fakt bleibt Fakt.	Look at Turkey. Look at all countries with a Muslim majority. Facts are facts.
So geht islamische Mobilmachung	That's how Islamic mobilization works
LÜGENPRESSE!!!11eins!!	LYING MEDIA!!!11one!! (note: might be irony)
**STRATEGY (Which argumentative means does the speaker use?)**	
opinion	
@jrg_o @AndreasZymny @marcusthomm1 @RegSprecherman muss aber auch sagen dass es den meisten egal ist was für eine Politik betrieben wird...	@jrg_o @AndreasZymny @marcusthomm1 @RegSprecherman also have to say that most people don't care what politics are pursued...
@AnneWillTalk @DietmarBartsch Sehr geehrte Frau Will ich bin von Ihrer Mainstream geprägten Moderation sehr enttäuscht.	@AnneWillTalk @DietmarBartsch Dear Miss Will I am very disappointed about your mainstream moderation.
@HeikoMaas Bleiben Sie standhaft. Politiker die aus Überzeugung handeln und nicht aus Koalitionsgründen sind mir sympathisch	@HeikoMaas Stay strong. Politicians acting on their beliefs and not based on coalition agreements are very likeable to me
information	
die bisherigen Muslim. Attentäter kamen aus Staaten die nicht auf der Liste stehen	all Muslim assassins so far came from countries not on the list
Laut CDU -geführten Innenministerium 83% der antisemitischen Straftaten von Rechtsextremen begangen @Epanastatis04	According to the ministery of interior under the CDU 83% of anti-Seminitic crimes were commited by right-wing extremists @Epanastatis04
(Link) ...Auf diesen Artikel bezog ich mich teilweise mit meinen Aussagen. Vielleicht überliefert er dir die Argumente etwas besser als ich es konnte.	(Link) ...With my statements I partially refered to this article. Maybe it conveys the arguments somewhat better than I was able to.
question	
Ich möchte bitte wissen woher das Foto von dem weinenden Mann kommt und wenn mögl. wen es zeigt.Danke schon mal vorweg :)	I please want to know where the photo with the crying man comes from an if poss. who it depicts.Thanks upfront :)

Warum reichen die Beleidigungstatbestände nach §§ 185 STGB nicht?	Why are the elements of an insult according to §§ 185 STGB (note: penal code) not sufficient here?
Wieviele Kinder haben Sie befragt und an welchen Schulen?	How many kids did you interview and at which schools?
consequences	
Dann zieht Ihr Euch aus den Waffenproduktionen zurück und glaubt nicht der USA alles. Euer Wachstum ist der Tod von Millionen.	Then withdraw from the weapon production and don't believe everything the USA says. Your growth is the death of millions.
wer grün wählt bekommt Merkel!	who votes green gets Merkel!
Diese Asozialen zerstören durch ihren Vandalismus die Existenzen zahlreicher Bürger. Schon mal an die Familien gedacht deren Autos brennen?!	Those social misfits with their vandalism destroy the livelihood of numerous citizens. Ever thought of the families, whose cars are burning?! (note: The post probably refers to left-wing riots during the G20 summit in Hamburg in 2017.)
correcting somebody	
Jetzt hatet doch nicht alle so rum! Ist doch schön dass sich mal jemand von der AFD der sogenannten "Lügenpresse" stellt anstatt sie 1/2	Please everyone, don't hate so much! After all, it is nice that someone from the AFD (note: Alternative für Deutschland, Alternative for Germany, German right-wing party) faces the "Lying Media" instead of 1/2
Ihre Position ist unfassbar und geschichtsvergessen. Den Tempelberg eine muslimische Stätte zu nennen ist antiisraelisch u. brandgefährlich	Your position is unbelievable and ignoring history. To call the Temple Mount a Muslim site is anti-Israeli and highly dangerous
Die Überschrift ist reine Irreführung und eine Frechheit der @tagesschau @ARD_Presse	The heading is pure deception and impudence of @tagesschau @ARD_Presse
inconsistency	
@RegSprecher Kanzlerin #Merkel's Sonntagsrede: „Der Kampf gegen Antisemitismus ist unsere gemeinsame Pflicht". Aber was tut sie? Nichts!	@RegSprecher Chancelor #Merkel's Sunday speech: "The fight against anti-Semitism is our joint duty." But what does she do? Nothing!
Böhmermann macht sich's leicht: Bei sich auf Kunstfreiheit pochen bei anderen aber das Sensibelchen spielen.	Böhmermann makes it easy for himself: Claiming freedom of art for himself and being sensitive when it comes to others.
@HeikoMaas Wer Menschen ihre eigenen politischen Überzeugungen absprechen will sollte sein Demokratieverständnis hinterfragen! @Craftergut	@HeikoMaas Someone who wants to deny people their own political convictions should quesion his understanding of democracy! @Craftergut
sarcasm	
Oh nein ein Politiker der an den Medien vorbei mit dem Volk kommuniziert! Wie pöse!	Oh no a politician communicating with the people without involving the media! How dare he!
Morddrohung wegen der Kopie eines Denkmals. Das sind wirklich sehr verletzende Steine.	Death threats because of the copy of a memorial. These really must be offending stones.

Ohne Kompromisse gehört er nicht in die Politik. Sollte sich bei Karl Lagerfeld bewerben.	Without being able to compromise he doesn't belong in politics. He should apply at Karl Lagerfeld.
personal insult	
ich muss kotzen bei Maas seiner HACKFRESSE	I have to vomit seeing Maas' SHITFACE
Der Söder hat exakt genauso viel Hirn wieder dreinschaut.	Söder has exactly as much brain matter as he looks.
Wie lächerlich dieser Satz von unser Schlaftablette Merkel wirkt.	How ridiculous this statement seems coming from our sleeping pill Merkel.
-isms insult	
ja dann zurück nach Afrika.Was erwarten die analphabeten hier zu finden? Die sollen ihre Länder aufbauen statt hier rum zu gammeln.	Then back to Africa.What do those illiterates expect to find here? They should build up their countries instead of hanging around here.
Übrigens Herr Maas ihr holt euch die grössten Juden-hasser ins Land und jetzt jammerst du rum!	By the way Mister Maas you are bringing the greatest anti-Semites into the country and now you are complaining!
Ist das wieder Werbung für den Drogen Beck und die geforderte unnütze Beauftragten Stelle? Ich mag Juden nicht und ihr werdet mir das nicht verbieten oder ändern! Sie sind Hauptverantwortliche für die chaotische Welt wie wir sie heute haben.	Is this advertisement for drug addict Beck and the demanded unnecessary commission again? I don't like the Jews and you will not forbid this to me or change me! They are the main responsible party for the chaos in the world as we know it today.
political insult	
Linke Abgeordnete! Scheinbar Mit-Finanzierer und Befürworter dieses Schleppertums!	Left-wing members of the parliament! Apparently co-funding and being in favour of this trafficking!
@tagesschau Das Gekreische der Linksextremen und Buntmenschen ist nur noch lächerlich!	@tagesschau The screaming of left-wing extremists and diverse do-gooders is just ridiculous!
Er wird nicht gewählt weil er ein AfDler ist. Egal wer von dem Kasperverein vorgeschlagen wird. Niemand will einen AfDler.	He won't be elected because he is from the AfD (note: Alternative für Deutschland, Alternative for Germany, German right-wing party). No matter who will be proposed from this club of clowns. Nobody wants someone from the AfD.
institutional insult	
Merkel hat das zu verantworten!Eu zeigt sein wahres Gesicht!Diktatur!	Merkel is responsible for this!Eu shows its true face!Dictatorship!
Keiner braucht die Propagandasender. Nur noch Hetze u. Lügen von ARD & ZDF	No one needs the propaganda stations. Only hate and lies from ARD & ZDF (note: the two biggest public TV stations in Germany)
Der Staatsfunk trommelt für eine GROKO	The state media cheers for the GROKO (note: GROße KOalition = large coalition; governing coalition of the two biggest parties in Germany CDU & SPD)
other	
@KiPos_info Da bei dir ein LTE-Mast steht bist du versorgt :P	@KiPos_info There is an LTE-mast near you, so you are covered :P
@janboehm herrlich!!! ROFL @neomagazin	@janboehm geart!!! ROFL @neomagazin

@janboehm @Lenas_view @welt In Israel isst man sehr gut arabisch (außer koscherdass ist gewöhnungbeduerftig)	@janboehm @Lenas_view @welt In Israel you can eat very good Arab food (except kosheryou need to get used to)
uninterpretable	
Kann mir jemand erklären wie ich als #e-Auto Besitzer die #Pkwmaut von der KFZ Steuer erstattet bekommen wenn ich keine zahle?	Can someone explain to me how I as an #electric-car owner can get reimburse for the car #toll within motor vehicle taxes if I don't pay any? (note: could be irony or an honest question)
TV-Skandal: Sender inszeniert Fluthilfe mit Flüchtlingen	TV scandal: broadcaster stages flood aid with refugees (note: could be an institutional insult if false or information if true)
Also bis auf die die zwischen 1933 und 1989 an den Folgen gestorben sind.	Except for those who died from the consequences between 1933 and 1989. (note: could be information, sarcasm, or an opinion)
**GROUP & GOAL (Which group is addressed in the tweet and what is the apparent goal of mentioning it?)**	
outgroup + threat	
Herr Maas wie ich es Ihnen schon mehrfach bewies Sie sind ein Problem für unsere Sicherheit und einem Rechtsstaat Ihr Unfähigkeit.	Mister Maas how I already proved multiple times to you you are a problem for our security and a constitutional state your incompetence.
Der familiennachzug wird unser Ende sein von dem wie wir Deutschland im Gedächtnis haben ! Das Ende der deutschen Kultur und Lebensweise !	Joining refugee's families will be the end of Germany as we know it ! The end of German culture and way of life !
30.000 beteiligtejeder 2. mit verbotene Flagge und die Polizei will und glauben lassen das sie Strafrechtlich verfolgt werden ?	30,000 people involvedevery 2. with forbidden flag and the police wants to make us believe that they are prosecuted ?
outgroup + weak	
Die lebt doch schon lange in ihrer eigenen bunten Welt. Kann im Duett mit Pippi #Nahles auftreten.	She lives in her own muddled world for a very long time already. Can sing a duet with Pippi #Nahles. (note: high-ranking German left-wing politician; her first name is not Pippi)
Die Politik sollte sich besser darauf konzentrieren diese Kulturbereicherer alle wieder loszuwerden	Politics should rather concentrate on getting rid of all those culture enriching people again.
Eine AfD ohne Rechtsextreme?	AfD without right-wing extremists? (note: Alternative für Deutschland, Alternative for Germany, German right-wing party)
ingroup + strengthen	
Egal welche Verbrechen ihr noch unterstützt das deutsche Volk wird kommen um es mit den Worten von Anonymous zu sagen "expect us"	No matter which crimes you are still supporting the German people will come to use the words of Anonymous "expect us"
Ein brillanter Kommentar! Endlich mal Klartext über die Zustände in Saudi-Arabien wie wir sie bei Amnesty seit Jahren beklagen. Danke!	A brilliant commentary! Finally an honest piece about the situation in Saudi-Arabia that we already denounce for years at Amnesty. Thanks!

Und wegen sowas braucht es eine Impfpflicht für alle insbesondere für Kinder.	And because of that we need compulsary vaccination for everyone especially for kids.
ingroup + justify	
@tagesschau Schon mal das Gesetz zum Schutz der Grenzen gelesen??Würde ich mal tun!!Sie zitiert nur geltendes Gesetz!!!	@tagesschau Ever read the law for the protection of borders??Would do!!She only cites law in force!!!
... moechte nicht sehen dass mein Staat auseinanderfaellt. Und werde sicherlich alles tun was ich nur kann um es zu vermeiden	... don't want to see how my country falls apart. And I will surely do everything in my power to prevent that
aber allen Deutschen über Generationen pauschal den Holocaust ständig vorwerfen? berechtigte Kritik oder Angst um Familie = Nazi... @omg	but generlly blaming all Germans over generations for the Holocaust? justified criticism or fear for your family = Nazi... @omg
both + common ground	
Ich empfehle die Lektüre unserer Verfassung. Die gilt für alle.	I recommend reading our constitution. It holds for all of us.
Ich war von eurer und unserer Politik heuer auch so früh erschöpft wie lange nicht mehr.	I was also exhausted from your and our politics as early this year as I haven't been for a very long time.
Jeder der so eine Tat begeht ist Geistesgestört! Da ist es egal zu welcher Religion oder Nation er gehört!	Everyone who commits such a crime is mentally disturbed! It doesn't matter to which religion or nation he belongs.
both + common problems	
Nicht Terrorismus Gleichgültigkeit die zur Entstehung der Flüchtlinge weltweit geführt hat ist Feind Nr 1.	Not terrorism indifference that is the origin of refugees worldwide is enemy No 1.
Nicht nur bei Nachrichten. Wir bräuchten insgesamt mehr Gebärdensprache im TV	Not only for news. We need more sign language in TV in general
Uns ist es schon bekannt. Jetzt müsste sie endlich dafür sorgen das alles getan wird die Ziele zu erreichen. Autoindustrie und Kohlekraftwerke sind nur zwei Beispiele	We know this already. Now she finally has to take care that everything will be done to reach the goals. Automotive industry and coal-fired power plants are only two examples
neutral + not applicable	
Dann würden viele Menschen viele fertig Produkte nicht mehr essen.	This way a lot of people wouldn't eat many convenience products anymore.
Russland war noch nie aus der Kriese draußen. Aber die Sanktionen zeigten das freier Handel zerbrechen kann.	Russia was never out of crisis. But the sanctions showed that free trade can break down.
Wieviele Personen sind denn befragt worden?	How many people were questioned?
uninterpretable + not applicable	
wir sprechen von 49 Monatsgehältern eines Arbeitslosen die keine Motivation sind. Wieso dann einen Job suchen?	we are talking about 49 monthly salaries of an unemployed person that aren't a motivation. Why looking for a job then? (note: We don't know whether the writer affiliates with unemployed people or not.)
Deshalb #Erdogan deshalb #islam. #EU =ChristenVereinigung.	Therefore #Erdogan therefore #islam. #EU =Christianassosiation. (note: We cannot know whether the writer affiliates with Turkey or the EU.)

@AnkeJulieMartin @tagesschau @MDRINFO @KerstinPalzer BTW zeigt ihr Statement deutlich wie negativ u. vorurteilbehaftet der Begriff ist.	@AnkeJulieMartin @tagesschau @MDRINFO @KerstinPalzer BTW your statement shows clearly how negative a. prejudiced the term is. (note: We do not know whether the writer agrees or disagrees with the statement and hence which side they are on.)

S3.7 Interrater reliability

Interrater reliability on the development and test set

We used Krippendorff's alpha [13] to calculate interrater reliability (IRR). Table S3 shows interrater reliability for the development set (used for the set up of the classification scheme, relabeled afterwards and as first training data for the preliminary classifier for sampling), and the test set (independently labeled batch for IRR calculation and final classifier validation). Krippendorff's alpha generalizes to an arbitrary number of coders and adjusts for the success of agreement by chance, i.e., the base-rate of the classes within a category. This feature is especially desirable in our context, where some classes occur much more frequently (e.g., "opinion") than others (e.g., "consequences").

Table S3: **Krippendorff's alpha values of interrater reliability**. Values are displayed for batch 1 (development of the classification scheme) and batch 2 (test batch). *Note:* [a] between two annotators [b] between four annotators.

data set	HATE	TARGET	STRATEGY	GROUP	GOAL
development[a]	.52	.53	.41	.50	.47
test[b]	.50	.58	.45	.42	.45

Interrater reliability does not meet the recommended standards by Krippendorff (>.667 for acceptable IRR, >.8 for good IRR [14]). Importantly, we expected to reach only moderate levels of interrater reliability due to the characteristics of the labeling task. Tweets are restricted in length and contain internet slang making them ambiguous by nature. That said, often times more context was needed to interpret the content and meaning of a tweet reliably. Furthermore, we allowed only one label per labeling category and tweet in order for the data to be compatible with classifier training later in the process. It is intuitive, however, that a single tweet can contain a mixture of argumentation strategies or socio-psychological goals. For example, a speaker could use pointing out inconsistencies as a mean to support their opinion. Similarly, whether a tweet is perceived as hateful can not only depend on (missing) context, but also on the reader [15, 16]. In those cases, we instructed our annotators to choose the class they perceived as dominant in a tweet to agree on a single label. Lastly, interrater reliability is not to be confused with the validity of the classification scheme. We attempted to capture the complexity of the problem without providing oversimplified rules to boost interrater reliability. We tried to disentangle some of the ambiguity of the labeled data with sophisticated classifier training techniques (e.g., training on confident samples in a staged

training process; see Section S4.2 for further details). In line with that reasoning, extant studies using human labeling of online content reached similar levels of Krippendorff's alpha (.39 in [11], .45 in [12]).

Interrater reliability at annotator change

After labeling the first half of the dataset, one of the initial three annotators had to drop out due to other obligations, and another equally qualified annotator was brought in. The author in charge of the labeling (AH) took over the training of the fourth annotator during their onboarding process. As soon as adequate interrater reliability between the former annotators and the fourth annotator was reached (judged by the agreement in the development and test batch), the new annotator took over regular labeling tasks. At that time, Krippendorff's alpha values on three samples with $n = (100, 50, 50)$ tweets drawn from previously labeled data except the test batch including old and new annotators measured:

- $\alpha_{\text{HATE}} = (.39, .54, .38)$
- $\alpha_{\text{TARGET}} = (.74, .70, .72)$
- $\alpha_{\text{STRATEGY}} = (.41, .35, .39)$
- $\alpha_{\text{GROUP}} = (.46, .32, .37)$
- $\alpha_{\text{GOAL}} = (.56, .51, .42)$.

Extension of the test set for dimension GOAL

We extended the test set for GOAL, since it included too few examples of the class "inclusionary about in/both groups" to reliably evaluate the classifier. We asked two annotators to independtly label another random sample of 200 tweets biased towards this minority class on the GOAL dimension. Krippendorff's alpha between the two annotators on this data set was 0.44, in line with the interrater reliability values observed for the other labeling tasks (see Tab. S3).

Tracking interrater reliability along the annotation process

Figure S2 shows the Krippendorff's alphas over different batches of the annotation process, calculated using the tweets labeled by more than one annotator.

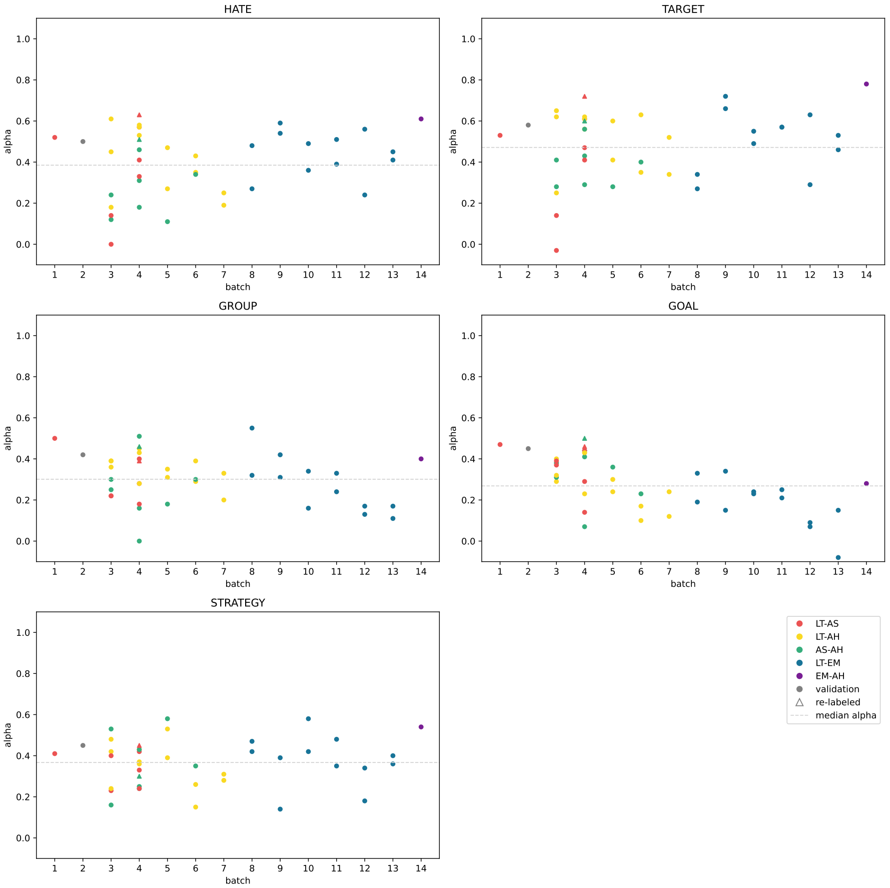

Fig. S2: **Krippendorff's alphas over different batches along the annotation process**. Different colors indicate different combinations of annotators in cross-labeling (legend denotes annotators' initials). Batches shown as triangles represent re-labeled batches after quality assurance measures, i.e. additional annotator conferences. The dashed line is the median over all alpha measures and should not be confused with alpha of the test batch (grey dot).

S3.8 Merging classes

Since the development of the classification scheme described in Section S2 was done in a classifier-agnostic and predominantly theory-driven way, some classes were extremely rare in the labeled training data set. For these classes, there were not enough diverse examples to enable the classifier to learn their characteristics and reliably generalize beyond the training data. In addition, tweets labeled as “foreign” (i.e., containing non-German language) were dropped from the training data set and a separate twitter-xlm-roberta-base model fine-tuned on the “foreign” labels to detect foreign language tweets was used to identify all other non-German tweets in the remaining corpus and remove them, too (see details on the model used in Section S4). Therefore the class “foreign” was also removed from the classification scheme going forward.

In order to improve classifier performance while still preserving the richness of information present in the human-annotated data, we merged some classes of the original classification scheme (see Tab. S1, column two) into larger classes, taking into account the frequency of classes and the similarity of the concepts they capture. Specifically, for the argumentation strategy (STRATEGY), we define the merged superclasses “constructive” (including original classes “information”, “question”, “consequences”, “correcting somebody”, and “inconsistency”), “leave fact” (i.e., leaving factual discussion, including the original classes “personal insult”, “-isms insult”, “political insult” and “institutional insult”), and “other” (including the original classes “uninterpretable” and “other”). The original classes “opinion” and “sarcasm” remained unchanged.

For in-/outgroup thinking (GROUP), we preserved “out” as the most frequent original class, and merged all other classes into the new superclass “not out” (including original classes “ingroup”, “both”, “neutral”, and “uninterpretable”).

For socio-psychological goal (GOAL), we created the new superclasses “exclusionary statements about outgroup” (including original classes “threat” and “weak”) and “inclusionary about ingroup or both groups” (including original classes “strengthen”, “justify”, “common ground”, and “common problems”). We preserved the original class “other” if GOAL was not applicable. Weakening the outgroup was by far the most prevalent socio-psychological goal, while more benevolent goals were very rare in the conversations.

Classes for hate speech (HATE) stayed as is, with labels “yes” and “no”.

S4 Training the machine learning model

S4.1 Masked language modeling

We used the pre-trained multilingual language model "twitter-xlm-roberta-base" [17] to develop classifiers by fine-tuning the pre-trained models on the human annotated training data sets. Before fine-tuning the model on the classification tasks, we pre-trained the model further with a masked language modeling (MLM) task on the full corpus of 1,167,853 tweets in the data set (domain-adaptive pre-training [18]). MLM is a self-supervised task and trains a model to predict a token that has been replaced with a "[MASK]" placeholder given its surrounding context. The goal of this masked language modeling is to improve the general performance of the model in the domain of interest to predict the next token given a series of tokens. We performed masked language modeling over 100 epochs, using a randomly selected sample of 20% of the corpus as validation set. MLM was performed with the following set of parameters: a learning rate of $2 \cdot 10^{-5}$, a weight decay of 0.01, 8 gradient accumulation steps, a batch size of 64, and a masking probability of 15%. Training performance was evaluated every epoch. The MLM task took 142 hours to complete on a single NVIDIA Quadro RTX 8000 GPU with 48 GB GDDR6 memory and reduced model perplexity on the validation set to 6.05.

S4.2 Fine-tuning and data augmentation

For fine-tuning, the classification head of the model was randomly initialized and then trained to classify tweets according to the merged classes in the classification scheme (see first column in Tab. S1). We fine-tuned a separate and independent classification head for each of HATE, STRATEGY, GROUP and GOAL, resulting in four distinct models. Each model was then used to predict the label of HATE, STRATEGY, GROUP and GOAL for every tweet in the corpus.

Even after our efforts to bias the selection of examples included in the training data set (see Section S3.3), we encountered severe class imbalances, where one class occurred frequently (majority class) and other classes were rare (minority classes). This made fine-tuning difficult, as the classifiers were prone to ignore classes with a low number of examples during training. In addition, the interrater reliability in all human annotated data batches pointed towards substantial ambiguity when it comes to assigning a single label to a given tweet (see Section S3.7). Therefore, using data labeled by a single rater during training was prone to introducing a large amount of confusing (text, label) pairs that impair classifier learning. On the other hand, we wanted to make use of as much of the available human annotated data as possible to improve how well the classifier could generalize outside the training data set. We therefore followed a training strategy where we trained the classifiers in several stages, using increasing amounts of labeled data where annotators agreed on the label,

supplemented with augmented examples and labels inferred by preliminary versions of the classifiers. We describe this strategy below.

Excluding the human-annotated held-out test set (see Section S3.2), we had a total of 2,259 examples with two labels by human annotators. Note that this is not a multiple of 50 because not all annotators completed their last data batch due to time constraints. To train the initial version of each classifier (one for each of HATE, STRATEGY, GROUP and GOAL), we only used examples where both annotators agreed on the label (we call them "confident examples"). This resulted in the following number of confident examples: STRATEGY: 1,279, GROUP: 1,664, GOAL: 1,821, HATE: 1,754. We provide an overview over the number of examples included in each classifier training stage in Tab. S4.

We then generated additional (augmented) examples from the confident examples and added them to the training data set. The goal of this step was to create examples of classes that were underrepresented in the training data by creating variations of existing examples that have the same meaning but different phrasing. We made use of back-translation [19, 20], where a given text in some language is translated into another language and then back to the original language, frequently leading to a rephrasing of the text, for example via replacing individual words with synonyms. To this end, we used MarianMT models [21] to translate each confident example into a target language and back to German. We did this for all languages, for which a forward and backward translation model was available (Afrikaans, Arabic, Czech, Danish, English, Esperanto, Spanish, Estonian, Finnish, French, Hebrew, Hungarian, Italian, Norwegian, Polish, Ukrainian, Vietnamese). We dropped all direct translation duplicates and then calculated the cosine similarity between the translation and the original text. Examples with a similarity in the bottom and top 10th percentile were discarded to get rid of examples that were either too similar, adding no new information for the classifier, or too dissimilar, indicating a failed translation. For each minority class in HATE, STRATEGY, GROUP and GOAL, we then added augmented examples generated via back-translation to the training data set until we ran out of augmented examples or there were as many examples of the minority class as of the majority class.

Before fine-tuning any model, we always removed URLs from the tweet texts and lower-cased all text.

In addition to the confident examples that had two labels by human annotators, we had 12,008 examples with a single label by a human annotator. To make use of these labels for the further classifier training process, we predicted a label for each of the 12,008 examples with the aim of including additional examples where both the single human annotator and the predicted label agreed. We started with the model that was pre-trained with a masked language modeling task on the full corpus as described above. We then fine-tuned this model on the confident examples plus the augmented examples (generated as described above) with a supervised prediction task for HATE, STRATEGY,

GROUP and GOAL, respectively. For every example, each of the models outputs a list of probabilities p_i that the example belongs to a given class i, where $\sum_i p_i = 1$.

To find optimal hyperparameters for model fine-tuning, we performed a random search for the model fine-tuned on STRATEGY and used the thus found hyperparameters for the HATE, GROUP and GOAL models as well. We did not perform separate hyperparameter searches for each model due to the computational cost. The random search was performed in the following parameter space: learning rate: [$1 \cdot 10^{-5}$, $5 \cdot 10^{-5}$, $1 \cdot 10^{-4}$], weight decay: [0.001, 0.0025, 0.005], label smoothing factor: [0.1, 0.2, 0.3], training batch size: [32, 64, 128, 256, 512].

If not noted otherwise, we used the following set of model parameters found via the hyperparameter search for each supervised prediction task described in the remainder of the section: a learning rate of $5 \cdot 10^{-5}$, weight decay of 0.0025, a label smoothing factor of 0.2 and a training batch size of 256. Evaluation was performed every 5 training steps with the macro-F1 score as the evaluation metric. Fine-tuning was done for a maximum of 10 epochs with early stopping after 5 evaluation steps with consecutively worse performance and 100 warmup steps. The maximum text length was set to 180 tokens.

Fine-tuning of the GOAL and HATE models was performed with a batch size of 128. Evaluation for these models was performed after every 10 steps (instead of every 5), to keep the number of examples the model sees between each evaluation step constant. To perform the fine-tuning, we used the "transformers" library for Python [22] (version 4.11.3).

To evaluate fine-tuning performance during training, we created five data splits for each model. Data splits were comprised of a training set (70% of the data), an evaluation set that was used to evaluate performance during training (15% of the data), and a validation set that was used to evaluate performance at the end of the training (15% of the data). Note that the final classifiers were validated against the held-out test set labeled by human annotators that we never used for fine-tuning (see Section S3.2 above and Section S5 below). Splits were created using scikit-learn's StratifiedShuffleSplit [23] function to create splits that preserve the percentage of examples for each class within the split. We therefore trained a total of five models for each of HATE, STRATEGY, GROUP and GOAL (one for each data split).

We then used the fine-tuned model with the best validation performance to predict labels for the 12,008 examples that only had a single human label. We compared the labels inferred by the classifier with the human labels and added examples to the training data where the model prediction and the human annotator agreed. Using this new training data set, we again created augmented examples for minority classes by back-translating the newly added examples and supplementing minority classes with augmented examples. We then used this new training data set to again fine-tune models for HATE, STRATEGY, GROUP and GOAL. This process was repeated for a maximum of two times

or until classifier performance did not improve anymore. In every data augmentation step, the models trained in the step before were used to predict labels in the remaining examples that only had one human label and adding examples where human and inferred label agreed to the training data set for the next data augmentation step. In each step we also added augmented examples of minority classes to improve class balance. An overview over the number of examples in the training data set for each model and class in each training iteration is given in Tab. S4. The table also indicates which data set was used to train the final version of the classifier for each of HATE, STRATEGY, GROUP and GOAL.

S4.3 Training data overview

As described in Methods, we use an iterative machine teaching approach [24] to train classifiers and augment training data. The first classifier version is trained only on examples where at least two human raters agree on the label ("confident labels"). We then add additional examples generated via back-translating the confident examples. We use this augmented data set to train a new classifier version that is used to infer labels for examples that only have one label from a human coder and add examples where the human coder and the inferred label agree (plus additional examples generated via back-translation) to the next version of the training data set. This process is repeated up to two times. Table S4 shows the number of examples for each class in each measure for dimensions and quality of discourse contained in the different versions of the training data set. The training data set version that was used for the final classifier, e.g., the classifier with the best performance on the held-out test set is indicated in bold.

category	class	confident labels	+ aug.	+ inf. & aug. (round 1)	+ inf. & aug. (round 2)
HATE	yes	1331	1331	**9120**	9495
HATE	no	423	830	**3839**	4205
TARGET	institution	96	188	**1149**	1247
TARGET	right-wing	62	123	**424**	483
TARGET	left-wing	55	110	**508**	784
TARGET	vulnerable	126	126	**1180**	1247
TARGET	other	9	18	**36**	50
STRATEGY	opinion	381	**391**	831	-
STRATEGY	constructive	199	**381**	1381	-
STRATEGY	sarcasm	122	**241**	257	-
STRATEGY	leave fact	336	**381**	412	-
STRATEGY	other	241	**381**	940	-
GROUP	not outgroup	679	985	3942	**4178**
GROUP	outgroup	985	985	3942	**4178**
GOAL	incl. abt. in/both groups	**248**	466	1226	-
GOAL	excl. abt. outgroup	**1088**	1088	1275	-
GOAL	other	**485**	950	1274	-

Table S4: **Number of examples for each class in different stages of classifier training**. Only labels where at least two human raters agreed ("confident labels"), added examples from back-translation ("+ aug."), examples where a human rater and the inferred label from a preliminary classifier agreed and additional examples from back-translation ("inf. & aug."). The training data used for the classifier with the best performance on the held-out test set is marked in bold letters. Note that for STRATEGY and GOAL we did not do a second round of inference and data augmentation, since classifier performance did not improve in the first round.

S5 Validation details

S5.1 Classifier performance on individual classes

The receiver-operating-characteristic (ROC) curves for individual classes are shown in Fig. S3 and the AUC values for every class are reported in Tab. S5. F1-score, precision and recall of the fine-tuned twitter-xlm-roberta models for individual classes of STRATEGY, GROUP, GOAL HATE, and TARGET are shown in Fig. S4.

Table S5: **Area under the curve (AUC) for every class**. ROC curves were calculated for each of the four models of HATE, STRATEGY, GROUP and GOAL. Reported AUC values are averages over the five ROC curves. Uncertainties are standard deviations.

model	class	AUC
HATE	hatespeech	0.87 ± 0.01
HATE	not hatespeech	0.85 ± 0.02
STRATEGY	constructive	0.83 ± 0.02
STRATEGY	opinion	0.84 ± 0.02
STRATEGY	sarcasm	0.78 ± 0.04
STRATEGY	leave fact	0.91 ± 0.01
STRATEGY	other	0.94 ± 0.01
GROUP	outgroup	0.88 ± 0.01
GROUP	not outgroup	0.88 ± 0.01
GOAL	inclusionary abt. in/both groups	0.79 ± 0.02
GOAL	exclusionary abt. outgroup	0.73 ± 0.01
GOAL	other	0.74 ± 0.01

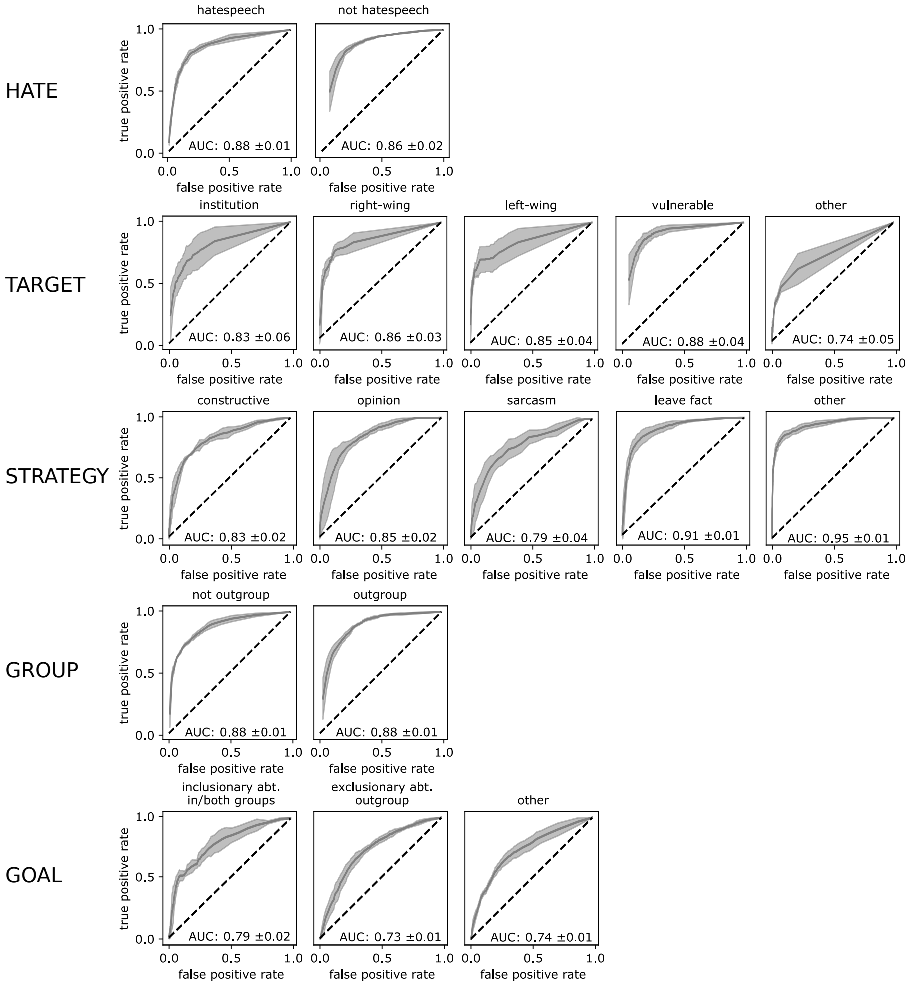

Fig. S3: **Performance of the fine-tuned twitter-xlm-roberta-base models**. Performance is depicted for models on HATE, TARGET, GROUP, GOAL and STRATEGY. For each class in every category we report the ROC curve and area under the curve (AUC). ROC curves and AUC are averaged over the performance of each of the five models trained on five different data splits. Shaded areas indicate standard deviations.

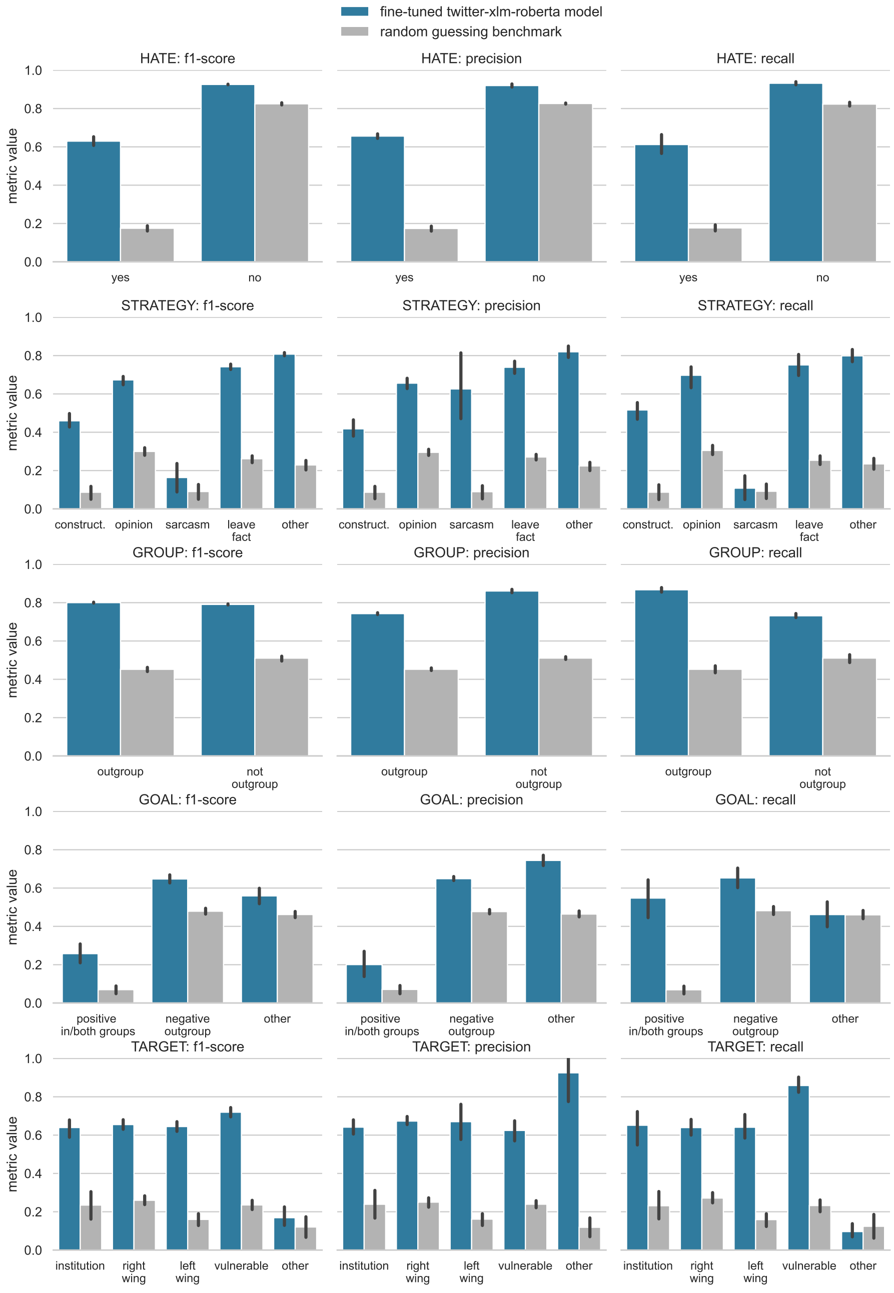

Fig. S4: **Performance of the fine-tuned twitter-xlm-roberta-base models**. Performance is depicted for models on HATE, TARGET, GROUP, GOAL and STRATEGY. For each class in every category we report the F1-score as well as precision and recall of the classifier (blue) compared to the performance of a frequency-based random guessing benchmark (grey). Performance metrics are averaged over the performance of each of the five models trained on five different data splits. Error bars denote the 95% confidence intervals.

S5.2 Determining classification thresholds for matching analysis

For the ARDL analysis described in Methods Section "Meso- and macro levels: Autoregressive distributed lag models" in the main manuscript, we did not need to binarize classes as we directly used the probabilities returned by the machine learning classifiers. For the non-parametric matching analysis described in Methods Section "Micro level: Causal inference at the level of individual reply pairs" in the main manuscript, for each tweet we needed to decide whether it contained a given class or not. To this end, we needed to find a threshold for the probability returned by the machine learning classifier above which an example would be labeled as a given class. To this end, we screened probability thresholds in increments of 0.01 to binarize the predicted labels in the human coded test set and then compared to the human labels. For each class, we then chose the threshold that maximized the F1 score (harmonic mean between precision and recall) to determine whether a given example belonged to a given class for the sake of the matching analysis. For measures with more than two classes, we first re-coded the labels in the human coded test set in a one-vs-many pattern for each class, therefore encoding examples of the class of interest as "1" while encoding examples from all other classes as "0". We report optimal thresholds as well as F1 scores, precision and recall in Tab. S6. Note that we did not follow this approach to validate our classifiers (see Section "Validation of machine learning classifiers" in the Methods of the main manuscript) since tuning thresholds against the human coded test set inflates performance metrics. For the classifier validation, we used the maximum probability returned by the machine learning classifier to label tweets.

For emotion classes, we determined the thresholds with the optimal F1-score using the data and materials distributed with the original article [25]. For the case of anger, we increased the threshold to 0.95 due to its prevalence in our dataset, as it constitutes a more contentious context than the one in which the emotion detection method was trained for.

category	class	opt. threshold	F1-score	precision	recall
HATE	yes	0.28	0.79	0.78	0.80
HATE	no	0.72	0.79	0.78	0.80
TARGET	institution	0.89	0.78	0.81	0.77
TARGET	right-wing	0.26	0.81	0.82	0.81
TARGET	left-wing	0.77	0.77	0.82	0.87
TARGET	vulnerable	0.97	0.83	0.82	0.84
TARGET	other	0.02	0.70	0.69	0.70
STRATEGY	opinion	0.40	0.78	0.79	0.78
STRATEGY	constructive	0.27	0.74	0.71	0.78
STRATEGY	sarcasm	0.28	0.67	0.66	0.68
STRATEGY	leave fact	0.47	0.84	0.84	0.84
STRATEGY	other	0.31	0.88	0.91	0.87
GROUP	not outgroup	0.41	0.80	0.80	0.80
GROUP	outgroup	0.59	0.80	0.80	0.80
GOAL	incl. abt. in/both groups	0.42	0.66	0.65	0.69
GOAL	excl. abt. outgroup	0.45	0.68	0.68	0.68
GOAL	other	0.31	0.70	0.70	0.70

Table S6: **Thresholds chosen to binarize class labels**. Binary labels were used for the matching analysis and associated F1-score, precision and recall.

S6 Descriptive statistics on the data set

The mean number of replies per user is 4.4 (interquartile range [0, 4]), with 51.3% of the users providing only a single reply (see Fig. S5A). The users that provided more than one reply are on average active for 358 days (interquartile range [43, 569]), considering their first and last post contained in our data set, which spans a total of 1,461 days.

"Root posts" of our discussion trees are somewhat equally distributed between the selected news outlets and journalists with most root users providing between 1,000 and 5,000 posts (see Fig. S5B). We note that the news outlets and journalists that provided the original "root" posts have been selected such that they persisted across time and with a focus on activity of hate and counter speech groups [4]. Root users represent major German news outlets or public figures, for example Twitter accounts of Germany's public-service broadcasters (ARD and ZDF).

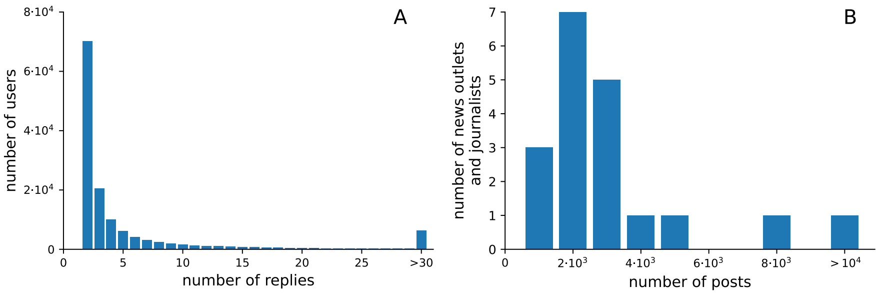

Fig. S5: **Reply and post statistics.** Left: Number of replies by number of users. Right: Number of posts by number of news outlets.

S7 Trends in dimensions and quality of discourse over time

Using a combination of human judgement and automated classifiers, we obtained measures of STRATEGY, GROUP, GOAL, HATE, and TARGET for each tweet in the 130,127 Twitter conversations sampled from the 1,461 days starting on January 1, 2015 and ending on December 12, 2018.

S7.1 Quality of discourse over time

For each tweet, hate speech, toxicity, and extremity of speech and speakers were measured on a scale from 0 to 1. For hate speech (HATE) and toxicity, higher values denote a higher probability that a human rater would perceive a tweet as hateful or toxic. For extremity of speech, higher values denote a higher probability that our ensemble classification system (see [4] and Section S1.2 for details) would label a tweet as representative of extreme political speech, exemplified either by the discourse of Reconquista Internet or of Reconquista Germanica. For the extremity of speakers, higher values denote a higher relative frequency of speakers whose tweets are labeled as containing extreme political speech (to be labeled extreme, the average extremity of one's tweets had to be higher than 0.7). As each trend has a different range of variation (see left panel of Fig. S6), for easier comparison we normalized them to the scale from their respective minimum and maximum values (right panel of Fig. S6). This makes it easier to see that all four trends follow similar trajectories, which is reassuring as hate speech, toxicity, and extremity measures are each based on different, independently developed classifiers (see Methods for details).

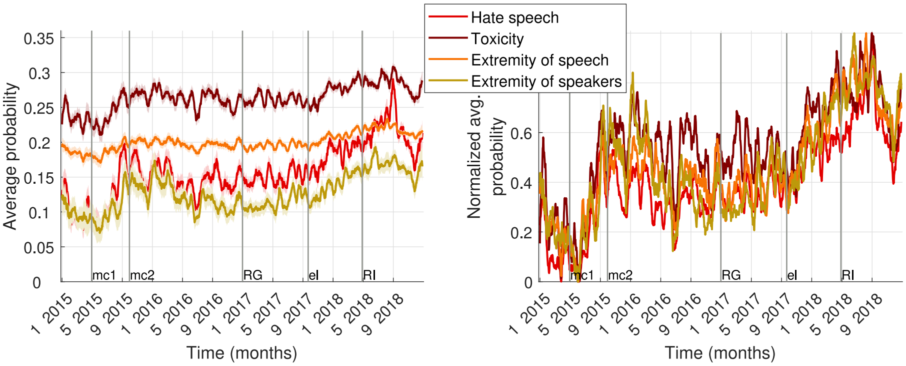

Fig. S6: **Indicators for quality of discourse over time**. Left: Raw values of each indicator, with standard error bands as rough indicators of variability. Right: Normalized values from minimum to maximum of each indicator, for easier comparison. Trends are smoothed over a two-week window. Thicker vertical lines denote several relevant events: mc1=beginning and mc2=peak of the migrant crisis, RG=start of Reconquista Germanica, el=2017 German elections, RI=start of Reconquista Internet.

Figure S7 shows further details about these dimensions and their relationships. The top-left panel shows that hate speech and toxicity have a monotonically positive relationship, with toxicity being particularly high (low) for tweets with the highest (lowest) probability of hate speech. The top right panel shows the political leaning of extreme speakers, depending on whether their tweets on average were similar to the discourse of Reconquista Germanica (RG) or of Reconquista Internet (RI). In this corpus, politically extreme speakers much more often use discourse typical of RG, likely leading to unbalanced political discussions. We see a small increase in the percentage of extreme speakers from the opposite side after RI was established, but overall these percentages are much smaller. The bottom panels show that toxicity and hate are not reserved for one or the other political orientation. Speakers and speech from both ends show higher levels of toxicity and hate than the more neutral speakers and speech. That said, the RG-like extreme of the political spectrum exhibits higher ratings of hate speech and toxicity than the RI-like extreme.

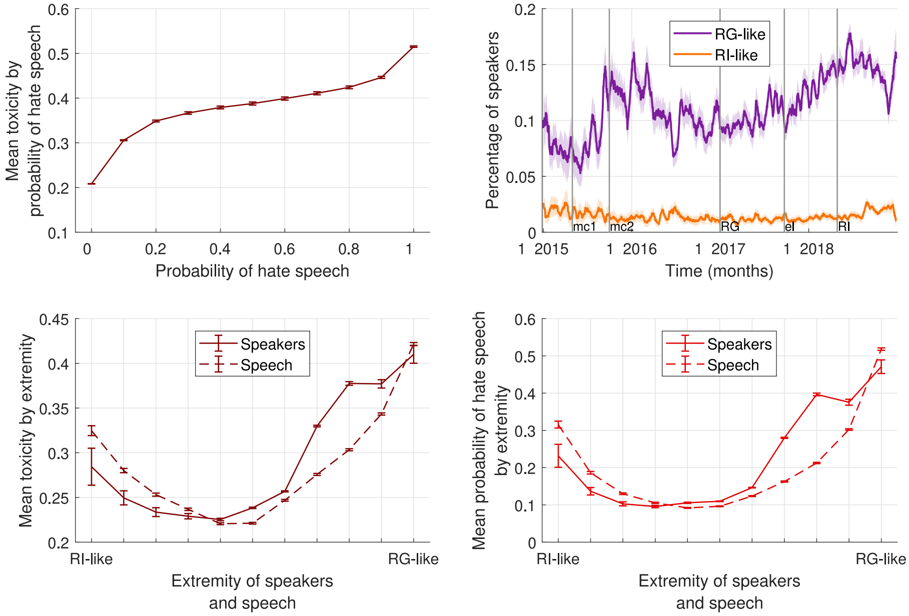

Fig. S7: **Relationships between indicators of quality of discourse**. Top left: Relationship between toxicity and probability of hate speech. Top right: Political leaning of extreme speakers (RG=Reconquista Germanica, RI=Reconquista Internet; error bands denote standard errors). Important events are marked as in Fig. S6. Bottom panels: Mean toxicity (left) and probability of hate speech (right) by different levels of extremity of speakers and speech. Trends are smoothed over a two-week window.

Figure S8 shows the average day-to-day probability that tweets containing hate were targeting specific groups. Vulnerable societal groups such as ethnic or religious minorities in Germany and institutions such as the German state or media outlets received a large part of all hate speech, with right-wing parties

receiving the least hate speech. The sharp increase in hate towards vulnerable groups coincides with the peak of the migrant crisis in Germany in the fall of 2015.

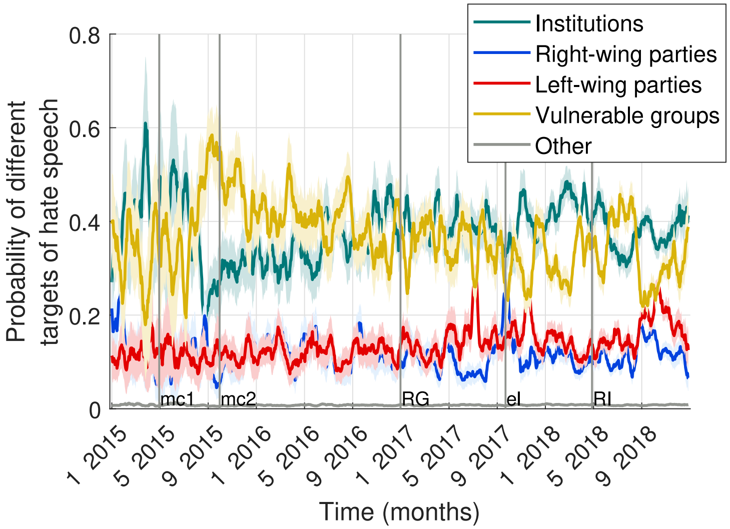

Fig. S8: **Targets of hate speech over time**. Trends are smoothed over a two-week window. Error bands denote standard errors. Important events are marked as in Figure S6.

Next, we show changes in the dimensions of discourse, which we will then use as predictors for quality of discourse described above. Each class within each of these dimensions is measured on a scale from 0 to 1, denoting the probability that a human rater would perceive that a tweet scores positively on a given class.

S7.2 Argumentation strategies over time

There is an increasing trend in expressing opinions—not necessarily objective, but without insults—as well as in the straightforward use of insults over time (Fig. S9). In contrast, the levels of constructive comments (providing information, asking honest questions, pointing out negative consequences, calling somebody out for behavior or choice of words, or exposing hypocrisy or contradictions) and sarcasm (name of the superclass including sarcasm, irony, and cynicism) have been decreasing slowly throughout the studied period.

We can also compare the use of different strategies in tweets that resemble the speech of the Reconquista Internet (RI, middle panel of Fig. S9) vs. tweets resembling the speech of Reconquista Germanica (RG, rightmost panel of Fig. S9). While the RG-like tweets more often contain constructive comments early on compared to the RI-like tweets, near the end of the time series they are more likely to contain insults than constructive comments.

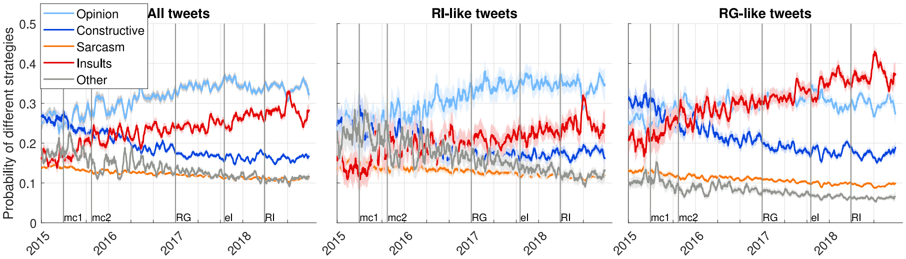

Fig. S9: **Probability of different argumentation strategies over time**. The panels show trends for all tweets (left), tweets similar to the speech of Reconquista Internet (RI, middle), and tweets similar to the speech of Reconquista Germanica (RG, right panel). Error bands denote standard errors. Important events are marked as in Fig. S6.

S7.3 Ingroup/Outgroup content over time

As described in Section "Classification scheme" in the main text, we discern whether a speaker referred to an ingroup or an outgroup in their tweet. These groups could be based on ethnicity (e.g., People of Color versus Caucasians), religion (e.g., Muslims versus Christians), attitudes (e.g., vegans versus meat eaters), or other characteristics. Over time, the content of discourse becomes more and more about participants' outgroups (Fig. S10). The outgroups were discussed particularly often on the right extreme of the political spectrum (in tweets classified as similar to the speech exemplified by RG).

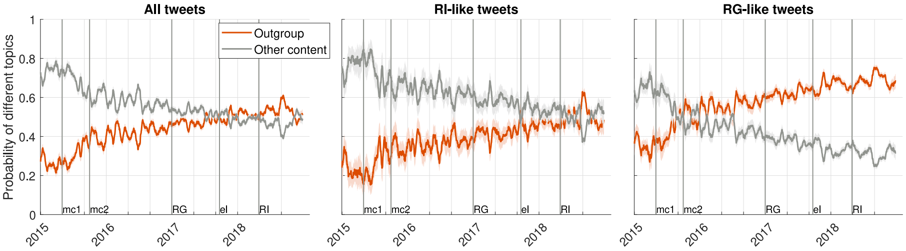

Fig. S10: **Probability of ingroup/outgroup content over time**. The panels show trends for all tweets (left), tweets similar to the speech of Reconquista Internet (RI, middle), and tweets similar to the speech of Reconquista Germanica (RG, right panel). Error bands denote standard errors. Important events are marked as in Figure S6.

The most frequent goal of tweets containing ingroup or outgroup content is excluding outgroups, while inclusionary statements about own or both groups are quite rare (Fig. S11). Both types of statements about own or both groups become more frequent over time. However, the ratio of these statements stays at roughly 2:1 over time for all tweets, and is even higher among tweets similar to the speech characteristic for Reconquista Germanica (the rightmost panel of Fig. S10).

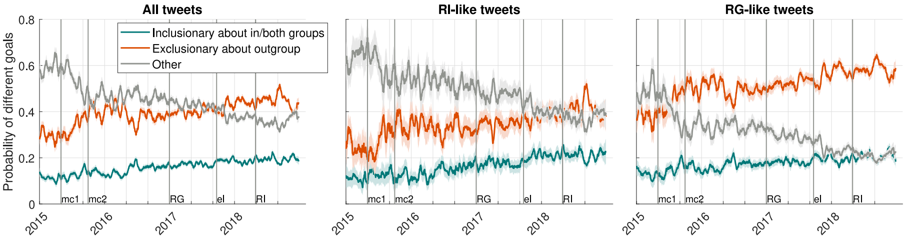

Fig. S11: **Probability of different goals regarding ingroup/outgroup over time**. The panels show trends for all tweets (left), tweets similar to the speech of Reconquista Internet (RI, middle), and tweets similar to the speech of Reconquista Germanica (RG, right panel). Error bands denote standard errors. Important events are marked as in Figure S6.

S7.4 Emotional tone over time

Finally, we explore the dynamics of tweets' emotional tone. As described in Section "Emotional tone" in Methods, emotions were determined independently of other dimensions of discourse, using a classifier that can detect multiple discrete emotions in German political text, published by Widmann and Wich [25].

The most prominent result is that anger dominates the emotional signature of the discourse in this corpus, followed by fear and sadness. The increase in anger echoes increases in toxicity, hate, and extremity over time (Fig. S6, as well as the increase in insults shown in Fig. S9) and exclusionary statements about outgroups (Fig. S11). This is reassuring from the methodological perspective as it shows that very different classifiers point to the same conclusions. Comparable to the previously shown trends, all negative emotions, including disgust, are more prominent in tweets that are similar to the extreme right speech as exemplified by Reconquista Germanica.

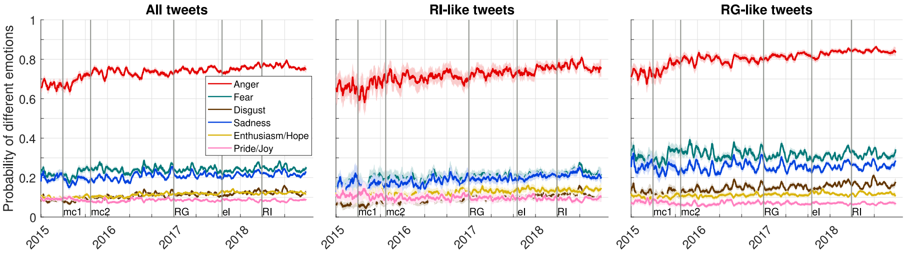

Fig. S12: **Probability of different emotions over time**. The panels show trends for all tweets (left), tweets similar to the speech of Reconquista Internet (RI, middle), and tweets similar to the speech of Reconquista Germanica (RG, right panel). Error bands denote standard errors. Important events are marked as in Fig. S6.

We also checked the correlations between different emotions (see Tab. S7). While the four negative emotions mostly correlate moderately with each other (around $r \in [0.3, 0.4]$, with the exception of sadness that correlates with fear and disgust at $r = 0.62$), the four positive emotions show two clear clusters:

enthusiasm and hope correlate highly with each other ($r = 0.83$) and joy and pride correlate with each other ($r = 0.67$), with significantly lower correlations between the clusters. This is partially in line with the results of [26] who found strong correlations between enthusiasm, hope, and pride. Our results establish pride as a separate construct from enthusiasm and hope, and are therefore more in line with [27] who stresses the role of group pride as an important and ubiquitous collective emotion. We use this reduced set of four negative and two positive emotions in the analyses reported in Fig. 2 in the main text.

S8 Relationship between discourse dimensions and quality

We use different machine learning classifiers to classify tweets for STRATEGY, TARGET, GOAL and GROUP. Machine learning classifiers return a probability that a given example belongs to a given class within these categories. For example, a tweet can at the same time have a probability of 0.87 of belonging to the class "leave fact" in STRATEGY, a probability of 0.6 of belonging to the class "left-wing" in TARGET, a probability of 0.55 of belonging to the class "outgroup" in GROUP, and a probability of 0.72 of belonging to the class "exclusionary about outgroup" in GOAL. In the following, we show scatterplots of the probability of belonging to a class in STRATEGY over the probability of belonging to a class in TARGET (Fig. S13), GROUP (Fig. S14) and GOAL (Fig. S15). In Figures S16 and S17 we show scatterplots of the probability of belonging to a class in GOAL over the probability of belonging to a class in TARGET and GROUP, respectively. We do not show scatterplots for other measures. Nevertheless, all predicted probabilities for dimensions and quality of discourse (including hate, toxicity and political extremity), as well as emotions are openly available under accession code 10.17605/OSF.IO/X4WE6 and can be explored using the raw data.

Correlation coefficients of all dimensions of discourse and measures for quality of discourse are shown in Tab. S7. Correlations are based on their average probability in each of the 1,150,469 tweets in our data set.

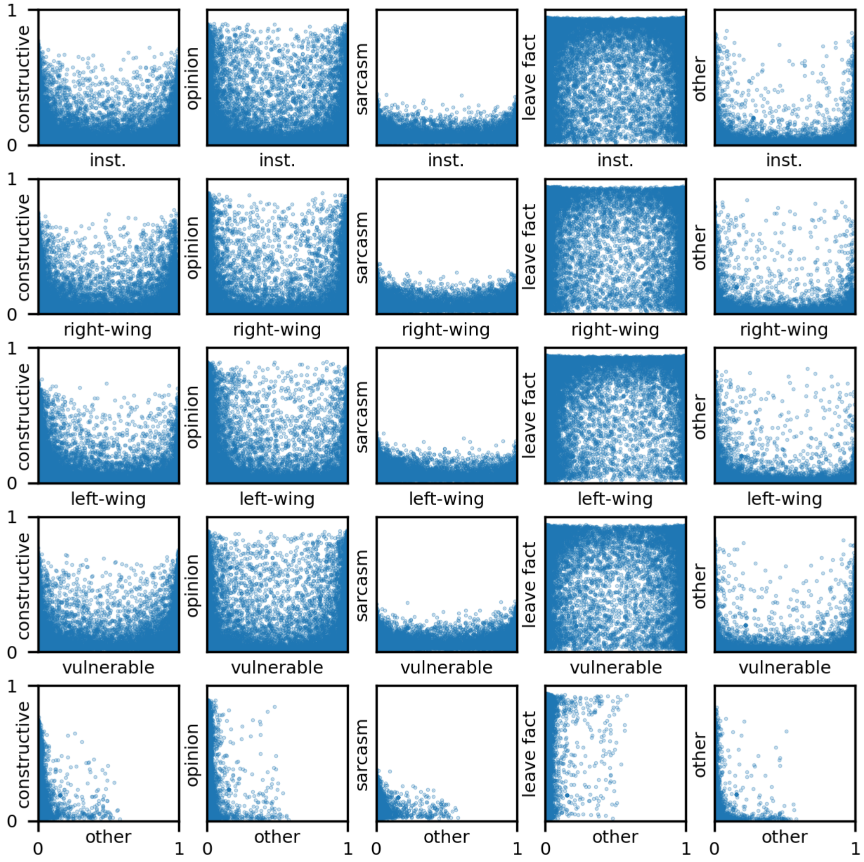

Fig. S13: Probability of different classes in STRATEGY over the probability of different classes in TARGET. *Note*: We only show a random sample of 25% of data points.

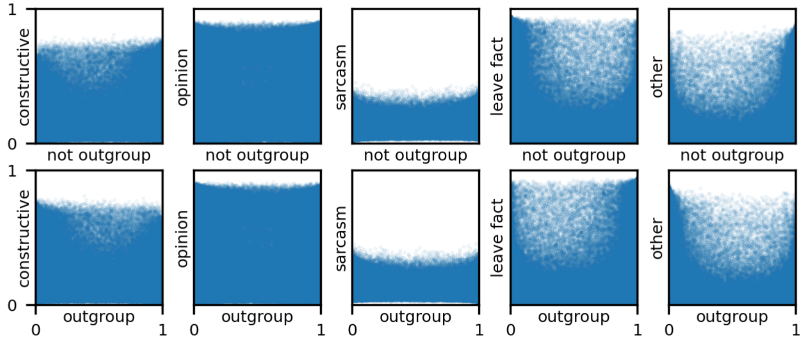

Fig. S14: **Probability of different classes in STRATEGY over the probability of different classes in GROUP**. *Note*: We only show a random sample of 25% of data points.

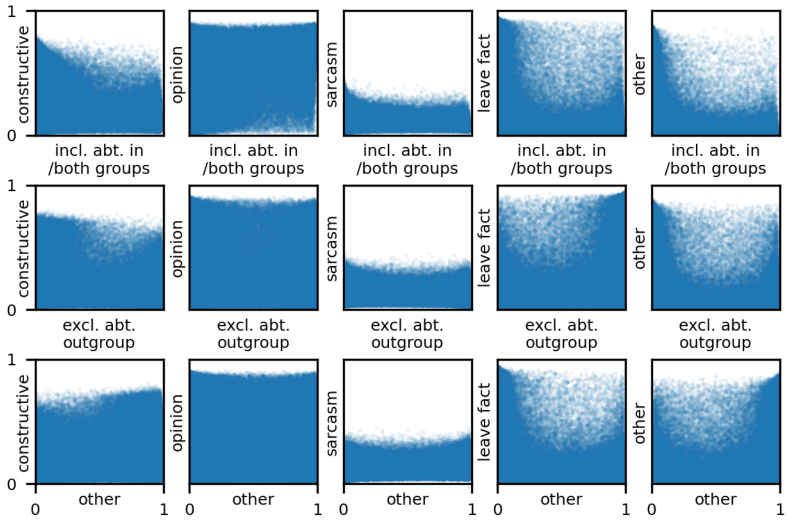

Fig. S15: **Probability of different classes in STRATEGY over the probability of different classes in GOAL**. *Note*: We only show a random sample of 25% of data points.

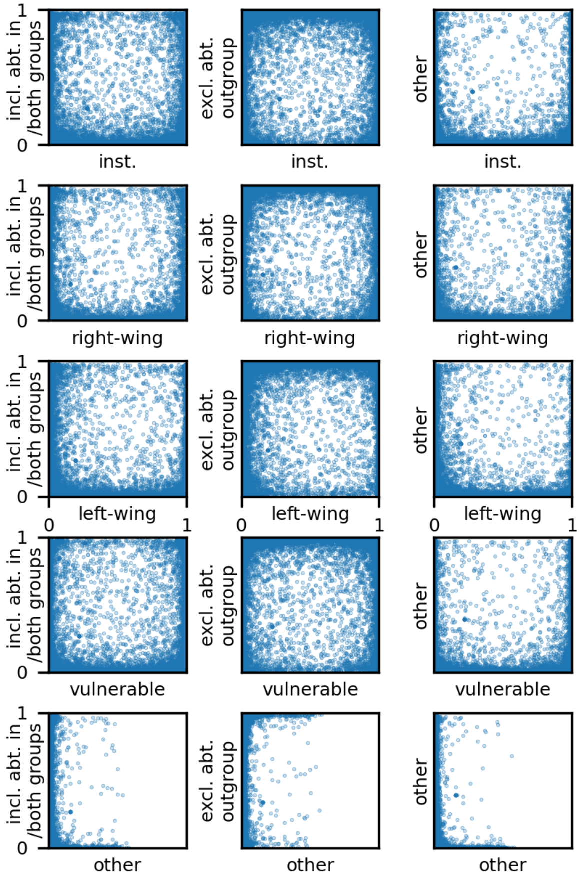

Fig. S16: **Probability of different classes in GOAL over the probability of different classes in TARGET**. *Note*: We only show a random sample of 25% of data points.

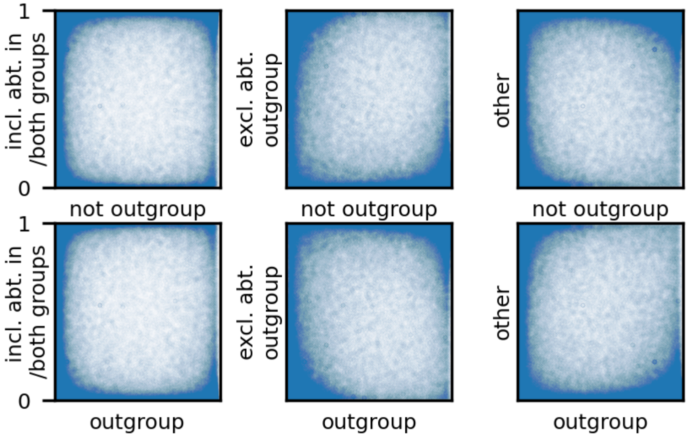

Fig. S17: **Probability of different classes in GOAL over the probability of different classes in GROUP**. *Note*: We only show a random sample of 25% of data points.

Table S7: **Correlations of different dimensions of discourse and measures for quality of discourse**. Correlations are based on their average probability in each of the 1,150,469 tweets in our data set.

**0**	**1**	**2**	**3**	**4**	**5**	**6**	**7**	**8**	**9**	**10**	**11**	**12**	**13**	**14**	**15**	**16**	**17**	**18**	**19**	**20**	**21**	**22**
**1 Hate speech**	1.00																					
**2 Toxicity**	0.42	1.00																				
**3 Speech**	0.25	0.19	1.00																			
**4 Speakers**	0.21	0.14	0.20	1.00																		
**5 Opinion**	-0.26	-0.24	-0.07	-0.04	1.00																	
**6 Constructive**	-0.17	-0.22	0.05	-0.07	-0.15	1.00																
**7 Sarcasm**	-0.34	-0.33	-0.15	-0.12	-0.18	0.33	1.00															
**8 Insults**	0.55	0.56	0.19	0.16	-0.56	-0.38	-0.47	1.00														
**9 Other**	-0.24	-0.24	-0.20	-0.09	-0.33	-0.18	0.37	-0.31	1.00													
**10 Outgroup**	0.47	0.53	0.23	0.16	-0.20	-0.25	-0.40	0.67	-0.43	1.00												
**11 Other content**	-0.47	-0.53	-0.23	-0.16	0.20	0.25	0.40	-0.67	0.43	-1.00	1.00											
**12 Incl. in/both**	-0.02	-0.10	0.02	0.02	0.50	-0.03	-0.16	-0.27	-0.20	-0.16	0.16	1.00										
**13 Excl. outgroup**	0.43	0.54	0.20	0.15	-0.31	-0.28	-0.37	0.74	-0.36	0.81	-0.81	-0.38	1.00									
**14 Other**	-0.43	-0.48	-0.23	-0.16	-0.03	0.31	0.49	-0.57	0.51	-0.72	0.72	-0.32	-0.76	1.00								
**15 Anger**	0.25	0.41	0.15	0.10	-0.11	-0.13	-0.21	0.40	-0.29	0.53	-0.53	-0.15	0.52	-0.43	1.00							
**16 Fear**	0.27	0.26	0.21	0.09	-0.02	0.14	-0.15	0.15	-0.28	0.23	-0.23	0.09	0.18	-0.25	0.38	1.00						
**17 Disgust**	0.30	0.50	0.15	0.10	-0.18	-0.08	-0.27	0.40	-0.23	0.40	-0.40	-0.12	0.40	-0.33	0.39	0.41	1.00					
**18 Sadness**	0.16	0.24	0.13	0.05	0.00	0.15	-0.13	0.09	-0.23	0.16	-0.16	0.07	0.09	-0.14	0.28	0.62	0.61	1.00				
**19 Enthusiasm**	0.02	-0.07	-0.02	0.01	0.27	-0.08	-0.11	-0.13	-0.08	-0.09	0.09	0.46	-0.16	-0.15	-0.35	-0.12	-0.22	-0.23	1.00			
**20 Hope**	-0.04	-0.15	-0.03	-0.01	0.31	-0.04	-0.03	-0.20	-0.07	-0.14	0.14	0.42	-0.21	-0.08	-0.36	-0.09	-0.32	-0.31	0.83	1.00		
**21 Pride**	-0.08	-0.15	-0.05	-0.02	0.17	-0.04	0.01	-0.17	0.07	-0.18	0.18	0.26	-0.21	0.04	-0.48	-0.30	-0.21	-0.20	0.51	0.29	1.00	
**22 Joy**	-0.12	-0.18	-0.08	-0.04	0.10	-0.04	0.10	-0.19	0.16	-0.23	0.23	0.09	-0.22	0.16	-0.52	-0.28	-0.19	-0.24	0.17	0.17	0.67	1.00

S9 Details of statistical analyses

We conduct analyses on three levels of discourse. On the micro level, we use matching analysis to determine how tweets affect directly subsequent tweets, enabling us to understand what might happen if people were actively using different discourse strategies to affect the content of direct replies to their tweets. On the meso level, we use ARDL models to analyze fine-grained discourse dynamics within discussion trees. On the macro level, we use those models to analyze day-to-day discourse dynamics, effectively summarizing the whole data set. Taken together, these three levels of analysis provide a nuanced picture of how different dimensions of discourse affect the quality of the subsequent discourse.

S9.1 Micro level: Causal inference at the level of individual reply pairs

Our analysis at the micro level (shown in panel A in Fig. 2 in the main text) is based on the identification of discussion acts in which user A writes a tweet, which is replied to by user B, followed by another tweet by user A in the same tree. This second tweet of user A could be in reply to user B or not - the only constraint is that there is no other tweet in the tree by user A after the reply written by user B. Our aim is to measure the effect of the discourse dimensions (argumentation strategy, ingroup/outgroup content, and emotional tone) in the reply written by user B on discourse quality (hate speech, toxicity, and extremity of speech) in the second tweet by user A. Note that we cannot analyze the extremity of speakers this way, because it would be determined by the identity of user A, which is incompatible with our covariate correction approach as explained below.

We determine whether the reply tweet contains a discourse dimension if the score of the corresponding classifier is above the threshold that leads to the highest F_1 score (see Section S5.2 in the SI for details), with the exception of anger, for which we set a higher threshold of 0.95 due to its prevalence in the dataset. We assign replies with a score above the threshold to the treatment group and replies with a score equal to or below the threshold value to the control group. Ideally, a randomized controlled trial would have assigned replies to the treatment and control groups completely at random, but as they happened in a natural setting, we cannot assume that treatments were randomly assigned. To approximate a random assignment, we apply nonparametric matching [28] to balance the treatment group with a subset of the control group for each discourse dimension. The matching assures minimal difference between the treatment and the control groups with respect to a set of covariates that could bias the content of replies. These covariates include scores on all discourse dimensions and discourse quality of the first tweet by user A, log-transformed variables counting the position of the tweet being replied to, the size of the tree, and other properties of user A such as their number of tweets in the whole dataset and the number of replies they have received.

This way, we correct not only for what kind of content attracts different types of discourse, but also for the prominence of discussions and the popularity of the user receiving the reply. We match the dataset with the MatchIt R library [29] using Mahalanobis distance and the nearest neighbors algorithm. A comparison of the matched variable averages before and after matching reveals satisfactory reductions of the standardized mean difference between groups to less than 0.1 for almost all variables and treatments. The only exception is the matching for the argumentation strategy "opinion", which has some remaining imbalance for opinion and inclusionary content scores, but with a standardized mean difference below 0.2 and substantially lower than the unmatched case.

After matching, we fit a linear regression model of the outcome score as a function of the treatment, including interaction effects and intercepts for all the covariates considered in the matching, i.e. double-adjusting [30] to correct for residual imbalances after matching. We add one more control variable to this model, namely whether the second tweet of user A was a reply to the reply by user B or not. We did not include this variable in the matching as it is part of the outcome and not one of the possible confounders of the treatment. We measure the causal effect of the treatment on the outcome as the marginal effect in this model with matching, calculated with the marginaleffects R library [31].

We calculate 95% confidence intervals of causal effects via bootstrapping on the matched sample including a propagation of the classification error of the treatment into the uncertainty of the estimate. We do so by applying a similar approach as [32], where we invert the classification error matrix in our tests of each classifier. This way we can calculate the rate of false positives and false negatives for each classifier, which we use to resample a simulated treatment variable as part of the generation of bootstrapping samples. This inflates confidence intervals and p-values by considering the measurement error, thus leading to conclusions that are robust to the noise introduced by machine learning classifiers. Furthermore, our analysis with dependent variables as classification scores rather than predicted classes is a way of accounting for measurement error in outcomes, both in this micro-level analysis and in the meso- and macro-level analyses using ARDL models, explained below.

S9.2 Meso- and macro levels: Autoregressive distributed lag models

To investigate the relationships between measures of discourse quality (hate speech, toxicity, and extremity of speech and speakers) and dimensions of preceding discourse (argumentation strategy, ingroup/outgroup content, and emotional tone), we used the autoregressive distributed lag (ARDL) modeling framework [33, 34], typically used for analyses of economic time series. For our purposes, this framework is interesting because it enables estimation of the effects of the dependent variable on itself, as well as direct and lagged effects of independent variables, in a single-equation form:

$$y_t = c_0 + c_1 t + \sum_{i=1}^{p} \varphi_i y_{t-i} + \sum_{i=0}^{q} \beta_i x_{t-i} + u_t \quad (1)$$

where c_0 is a constant, $c_1 t$ is a time trend, y_{t-i} are lags of the dependent variable y_t with associated weights φ_i denoting dynamic marginal effects of y on itself for p lags, x_{t-i} are lags of independent variables with the associated weights β_i denoting dynamic marginal effects of x on y for q lags, and where u_t is an error term. In this way, we can study each of the effects independently since weights for one variable are adjusted by the influence of other variables in the statistical model.

We test the assumptions for validity of all models using a variety of tests. First, we use Dickey-Fuller test [35] to examine the assumption, necessary for ARDL models, that all our variables are integrated of order 0 or 1 ($I(0)$ or $I(1)$) [34]. We find that this is the case for all predictor variables, and the dependent variables are all integrated of order 0 (for the variables included in the four macro-level models, see Table S8; results for the 3,569 meso-level models are equivalent and available on request). Given this, we do not explore the presence of cointegrating relationships with tests such as the bounds test [36].

Second, to determine how many lags have explanatory power for this data, we compare models with different numbers of lags using the AIC measure [33]. For all models, a maximum of two lags were sufficient to explain the relationship between our predictors (the dimensions of discourse) and outcomes (the quality of discourse).

Finally, after fitting the models, we examine the distribution of residuals and the associated homoskedasticity using White's chi-square [37]. We also check for the presence of serial correlation using the Breusch-Godfrey LM test for autocorrelation [38] and apply the cumulative sum test for parameter stability [39]. Most models showed satisfactory results on all tests, with the occasional borderline heteroskedasticity. We therefore calculate and report only robust standard errors for all results.

For each measure of discourse quality (hate speech, toxicity, extremity of speech and speakers), we apply ARDL models on two different levels of data aggregation. On the meso level, we aim to illuminate short-term dynamics within trees, by applying ARDL models over successive tweets in discussion trees that contained at least 50 tweets (panel B in Fig. 2 in the main text and Figure S19). To check whether longer discussion trees exhibit different discourse dynamics, we also replicate the analyses with 868 trees that include at least 100 or more tweets. Most patterns of results remain the same (see Results section in the main text and Fig. S21), although some patterns are more pronounced with more (albeit shorter) trees. We could not analyze even shorter trees because our models would become overspecified.

On the macro level, we aim to provide information about more general and longer-term effects on the discourse quality, by applying ARDL models over average measures of each dimension for each of the 1,461 days in our time

series (panel C in Fig. 2 in the main text and Figure S20). Note that these macro-level analyses include all tweets from all trees.

Because the number of predictors ARDL models can handle is limited (as each predictor is a complete time series), we had to refrain from including all potentially interesting predictors and their interactions. In particular, we could not include both insults and exclusionary language about outgroups, because of collinearity issues. We chose to include the exclusionary language but not insults for two reasons: to be able to compare its effects with those of the inclusionary language about ingroup or both groups; and because the insults strategy was conceptually very similar to the dependent variables of hate speech and toxicity, so including them seemed circular.

Furthermore, because one of our interests in this paper is exploring the effects of civic self-organization on discourse, we investigate how the presence of organized groups Reconquista Germanica (RG) and Reconquista Internet (RI) interacts with the effects of different discourse dimensions. Specifically, we use a metaregression of tree-level results to investigate i) the effect of the time period in which a discussion tree occurred, including dummy variables for the periods when RG was active (from January 2017 on), and when RI was also active (from May 2018 on), and ii) the relative proportion of tweets in a tree that were posted by users whose speech resembled either political extreme (that is, was similar to either RI or RG, as described before). As controls, we also include iii) the category of the account that posted the first tweet in a tree (media, journalist, or a politician), iv) the total number of tweets in a tree, v) the duration of the discussion in a tree in hours as measured by the time difference between the first and the last tweet contained in a tree, and vi) the number of unique participants in a tree. The only consistently reliable interactions were with variables ii) above, so we will discuss only those in what follows (see complete results in Section S12).

Similarly, for the day-to-day analyses, we included exogenous effects of the time periods when none of the organized groups were active, when RG was active (from January 2017 on), and when RI was also active (from May 2018 on). We include dummy variables for the latter two periods, as well as their interactions with all discourse dimensions as exogenous effects. To enable a quick overview of these results, we mark the direction of all reliable interactions with the extremity of speakers for the tree-level analyses, and with the overall presence of RG and RI on Twitter for the day-to-day analyses using icons resembling the RG logo (a sign that combines letters R and X and resembles a sword) and the RI logo (a sign that resembles a heart), respectively (see Fig. 2 in the main text, and Figs. S19 and S20). For example, if a dimension has an overall negative effect on hate speech, and the effect becomes even more negative when RG is active and/or present in a tree, then we add the icon for RG to the left of the effect. If the effect becomes more positive, we add this icon to the right of the effect; and we do the same for all reliable interactions with the presence of RI. All results are shown in detail in Section S12.

To implement the models, we use Stata ARDL package [34]. We re-checked our analyses using R ARDL package [40] and found that the results are comparable.

Table S8: **Results of Augmented Dickey–Fuller tests for all variables included in the macro-level models.**

	Direct effects			Lag 1			Lag 2		
	Coef.	SE	p	Coef.	SE	p	Coef.	SE	p
Hate speech	-0.297	0.025	<0.001	-0.262	0.029	<0.001	-0.161	0.026	<0.001
Toxicity	-0.353	0.028	<0.001	-0.249	0.030	<0.001	-0.149	0.026	<0.001
Extreme speech	-0.366	0.029	<0.001	-0.337	0.031	<0.001	-0.156	0.026	<0.001
Extreme users	-0.294	0.027	<0.001	-0.450	0.030	<0.001	-0.198	0.026	<0.001
Opinion	-0.331	0.028	<0.001	-0.332	0.030	<0.001	-0.183	0.026	<0.001
Sarcasm	-0.481	0.033	<0.001	-0.214	0.031	<0.001	-0.148	0.026	<0.001
Constructiv	-0.317	0.027	<0.001	-0.287	0.029	<0.001	-0.178	0.026	<0.001
Excl.outgroup	-0.346	0.027	<0.001	-0.265	0.030	<0.001	-0.130	0.026	<0.001
Incl. in/both groups	-0.508	0.033	<0.001	-0.209	0.032	<0.001	-0.120	0.026	<0.001
Anger	-0.433	0.031	<0.001	-0.291	0.031	<0.001	-0.129	0.026	<0.001
Fear	-0.475	0.031	<0.001	-0.185	0.031	<0.001	-0.076	0.026	0.004
Disgust	-0.434	0.030	<0.001	-0.160	0.030	<0.001	-0.106	0.026	<0.001
Sadness	-0.449	0.030	<0.001	-0.164	0.030	<0.001	-0.062	0.026	0.018
Enth./Hope	-0.551	0.035	<0.001	-0.196	0.032	<0.001	-0.107	0.026	<0.001
Pride/Joy	-0.627	0.036	<0.001	-0.132	0.033	<0.001	-0.026	0.026	0.323

S10 Relationship of discourse dimensions with quality of discourse

S10.1 Micro level

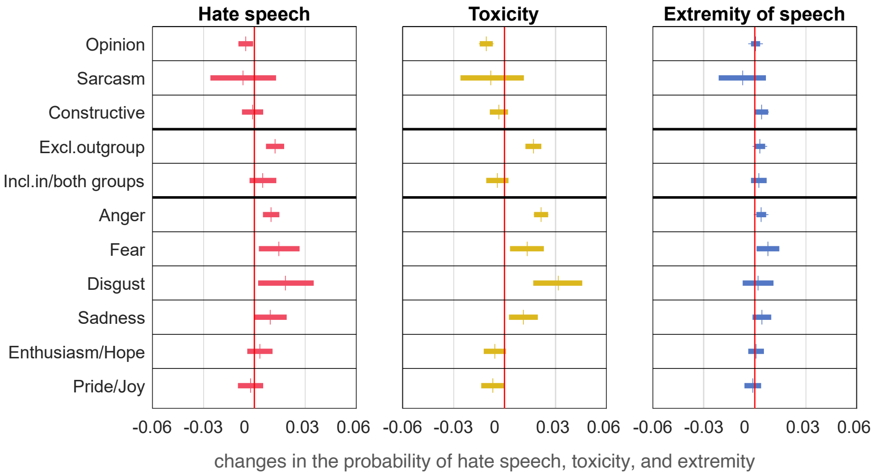

Fig. S18: **Micro-level effects on the probability of hate speech, toxicity and extremity, following tweets containing different dimensions of discourse.** The effects, obtained using matching analysis, describe discourse quality in second tweets of users who have received a reply (characterized by different dimensions of discourse) from another user to their first tweet (see S9 for details).

S10.2 Meso level

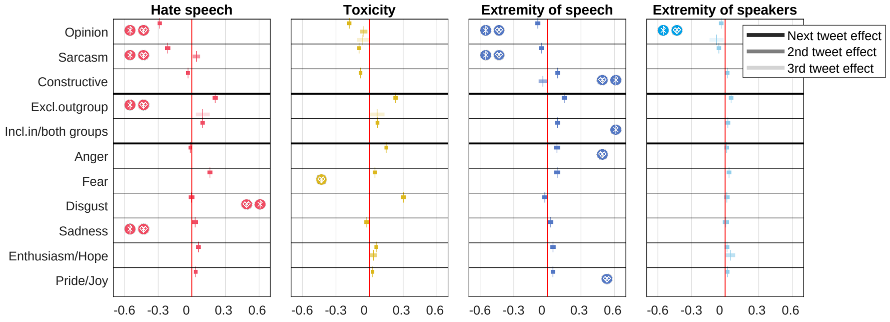

Fig. S19: **Meso-level effects on the probability of hate speech, toxicity and extremity in discussion trees, following tweets containing different dimensions of discourse.**. These effects within discussion trees were calculated as meta-analytic estimates from ARDL models fitted on 3,569 discussion trees (see S9 for details). Reliable lagged effects for the second next tweet were observed for 42% to 45% of trees and for the third tweet for 22% to 25% of trees; shown are averages of those reliably observed lagged effects. The icons of Reconquista Germanica (combined letters R and X resembling a sword) and Reconquista Internet (a sign that resembles a heart) denote the direction of reliable interactions with the percentage of extreme speakers resembling one of the groups in each tree. If an effect of a dimension became more negative (positive) when one or both of these groups were present, we added the respective icon to the left (right) side of the effect. Regression coefficients are provided in Table S13.

S10.3 Macro level

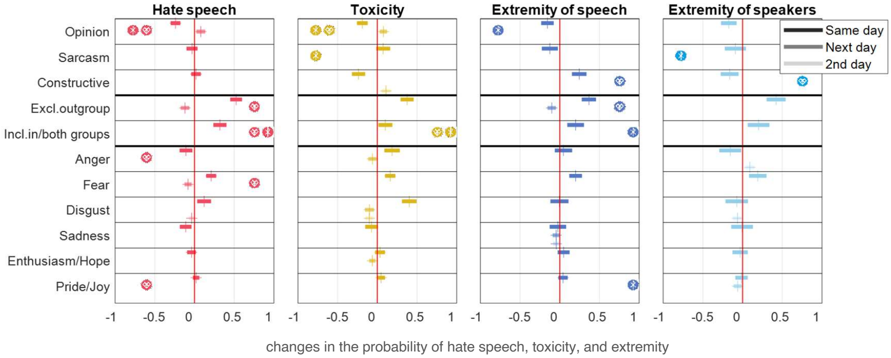

Fig. S20: **Macro-level effects on the probability of hate speech, toxicity, and extremity in days depending on the prevalence of different dimensions of discourse in the preceding days.** These effects were calculated as meta-analytic estimates from ARDL models fitted on averaged dimensions of discourse over each of 1,461 subsequent days (see S9 for details). The icons of Reconquista Germanica (combined letters R and X resembling a sword) and Reconquista Internet (a sign that resembles a heart) denote the direction of reliable interactions with the existence of one or both groups in the public sphere on a specific day. If an effect of a dimension became more negative (positive) when one or both of these groups were present, we added the respective icon to the left (right) side of the effect. Regression coefficients are provided in Table S14.

S11 Robustness analyses

In the following section we provide several analyses that demonstrate that the results presented in the main text are robust to various changes in the methodology. In Fig. S21 we show a comparison of estimates from the meta-regression of meso-level ARDL model estimates (influence of different discourse dimensions on discussion trees) obtained with reply trees of 50 or more tweets (analysis shown in the main text) and results obtained with reply trees of 100 or more tweets.

In Fig. S22 we show the estimates from the meta-regression of meso-level ARDL models of trees initiated by tweets from different types of users: prominent news organizations (2,630 trees), individual politicians (467 trees), and individual journalists and bloggers (562 trees).

Lastly, in Tables S9, S10, S11, and S12 we show an overview of meta-regression estimates of meso-level ARDL models for the hate speech, toxicity, extremity of speech, and extremity of speakers, respectively.

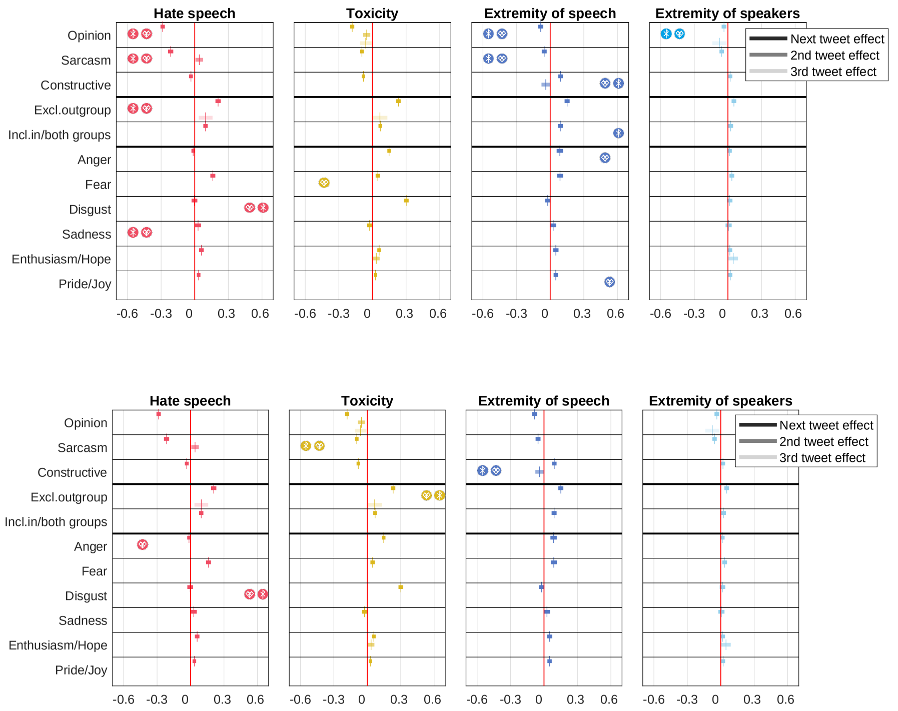

Fig. S21: **Comparison of meta-analytic estimates for the effects of different dimensions of discourse on changes in the probability of hate speech, toxicity, and extremity, for smaller and larger discussion trees**. Effects were obtained using ARDL models of 3,569 trees with at least 50 tweets (top row, also shown in Fig. S19 and partially in Fig. 2 in the main text) and 868 discussion trees with at least 100 tweets (bottom row). The patterns are similar, with some effects being more pronounced when calculated on smaller trees which are more numerous (top row).

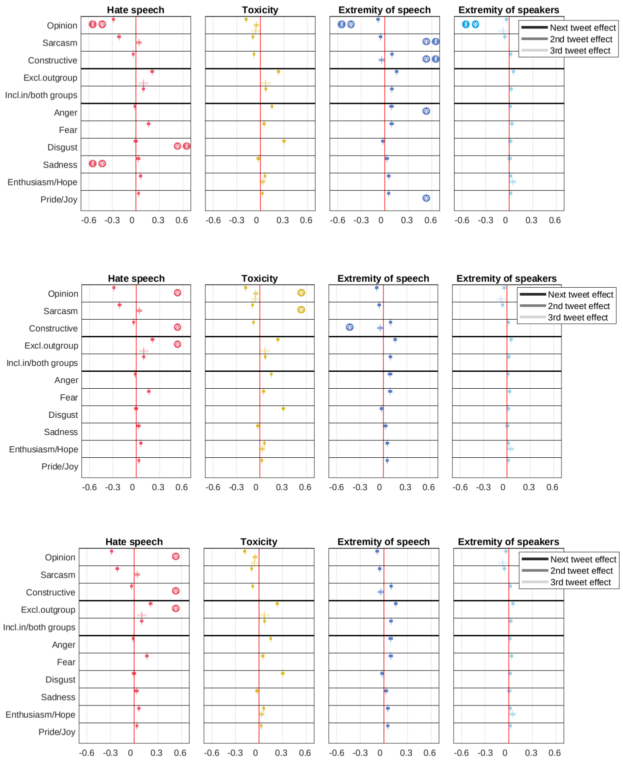

Fig. S22: **Comparison of meta-analytic estimates for the effects of different dimensions of discourse on changes in the probability of hate speech, toxicity, and extremity, separated by user type**. Estimates were obtained using ARDL models of trees initiated by tweets from different types of users: prominent news organizations (2,630 trees), individual politicians (467 trees), and individual journalists and bloggers (562 trees). Patterns of results are roughly similar for all three types.

Table S9: **Robustness analysis for discussion characteristics with respect to hate**. Meta-regression investigating how different properties of discussion trees affect the weighted average coefficients for Hate speech (as shown in the first column in Tab. S13).

		Opinion	Sarcasm	Constructive	Excl.out	Incl.in/both	Anger	Fear	Disgust	Sadness	Enth./Hope	Pride/Joy
RG	b	-0.024	-0.012	-0.001	-0.014	0.009	0.002	0.004	0.021	-0.022	0.006	-0.002
	se	0.006	0.006	0.005	0.007	0.006	0.005	0.007	0.008	0.007	0.006	0.005
	p	0.000	0.028	0.778	0.040	0.133	0.762	0.525	0.006	0.002	0.270	0.674
RI	b	-0.026	-0.015	0.009	-0.016	0.007	0.002	0.008	0.026	-0.021	0.007	-0.002
	se	0.006	0.006	0.005	0.007	0.006	0.005	0.007	0.008	0.007	0.006	0.005
	p	0.000	0.011	0.092	0.020	0.216	0.743	0.222	0.001	0.005	0.221	0.665
root: journalist	b	0.032	0.020	0.012	0.016	0.003	-0.006	0.004	-0.001	-0.006	-0.001	-0.002
	se	0.003	0.003	0.003	0.004	0.004	0.003	0.004	0.005	0.004	0.003	0.003
	p	0.000	0.000	0.000	0.000	0.405	0.043	0.309	0.817	0.161	0.737	0.491
root: politician	b	0.009	0.016	-0.008	-0.019	-0.009	-0.005	0.017	0.013	-0.002	0.006	0.001
	se	0.003	0.003	0.003	0.004	0.003	0.003	0.004	0.004	0.004	0.003	0.002
	p	0.004	0.000	0.003	0.000	0.007	0.078	0.000	0.001	0.573	0.049	0.829
tree length	b	-0.016	0.012	-0.025	0.013	0.017	0.001	-0.001	-0.019	0.006	-0.011	0.003
	se	0.006	0.006	0.005	0.007	0.006	0.005	0.007	0.008	0.007	0.006	0.004
	p	0.006	0.041	0.000	0.069	0.003	0.797	0.867	0.020	0.399	0.061	0.532
tree duration	b	0.004	-0.004	-0.002	-0.002	-0.002	-0.001	0.001	-0.003	0.001	-0.003	0.003
	se	0.003	0.003	0.003	0.003	0.003	0.003	0.004	0.004	0.004	0.003	0.002
	p	0.194	0.140	0.525	0.567	0.479	0.746	0.862	0.386	0.740	0.392	0.137
N unique speakers	b	0.019	-0.012	0.034	-0.017	-0.016	-0.001	0.011	0.022	-0.014	0.012	-0.002
	se	0.006	0.006	0.005	0.007	0.006	0.005	0.007	0.009	0.008	0.006	0.005
	p	0.003	0.042	0.000	0.024	0.009	0.850	0.131	0.012	0.078	0.046	0.683
% RG speakers	b	-0.007	-0.004	-0.007	0.021	0.022	0.001	0.018	-0.001	-0.007	0.010	-0.002
	se	0.003	0.003	0.003	0.004	0.004	0.003	0.004	0.005	0.004	0.003	0.003
	p	0.033	0.292	0.030	0.000	0.000	0.826	0.000	0.801	0.113	0.006	0.505
% RI speakers	b	0.007	0.007	0.012	0.019	0.011	0.002	-0.013	0.000	0.001	-0.003	-0.002
	se	0.004	0.003	0.003	0.004	0.004	0.003	0.004	0.005	0.004	0.004	0.003
	p	0.036	0.052	0.000	0.000	0.002	0.549	0.002	0.921	0.772	0.435	0.462
Constant	b	-0.344	-0.244	-0.025	0.173	0.104	-0.003	0.190	0.048	-0.005	0.079	0.033
	se	0.003	0.003	0.003	0.004	0.003	0.003	0.004	0.004	0.004	0.003	0.002
	p	0.000	0.000	0.000	0.000	0.000	0.357	0.000	0.000	0.170	0.000	0.000

Table S10: **Robustness analysis for discussion characteristics with respect to toxicity**. Meta-regression investigating how different properties of discussion trees affect the weighted average coefficients for Toxicity (as shown in the second column in Tab. S13).

		Opinion	Sarcasm	Constructive	Excl.out	Incl.in/both	Anger	Fear	Disgust	Sadness	Enth./Hope	Pride/Joy
RG	b	-0.006	-0.006	0.000	0.011	0.005	0.002	-0.010	0.007	-0.007	0.008	0.006
	se	0.005	0.005	0.005	0.006	0.005	0.005	0.006	0.007	0.006	0.005	0.004
	p	0.294	0.282	0.947	0.086	0.308	0.711	0.081	0.291	0.271	0.099	0.187
RI	b	-0.006	-0.003	0.000	0.008	0.008	0.005	-0.013	0.006	-0.004	0.007	0.003
	se	0.006	0.005	0.005	0.006	0.006	0.005	0.006	0.007	0.007	0.005	0.004
	p	0.265	0.570	0.978	0.228	0.129	0.373	0.026	0.349	0.504	0.203	0.556
root: journalist	b	0.011	0.007	0.006	0.003	-0.003	0.010	-0.003	-0.009	0.001	-0.002	0.002
	se	0.003	0.003	0.003	0.004	0.003	0.003	0.003	0.004	0.004	0.003	0.003
	p	0.001	0.026	0.028	0.467	0.324	0.001	0.389	0.020	0.850	0.569	0.475
root: politician	b	-0.001	0.000	-0.004	-0.004	-0.005	-0.001	0.000	0.003	-0.004	0.004	0.003
	se	0.003	0.003	0.002	0.004	0.003	0.003	0.003	0.003	0.003	0.003	0.002
	p	0.704	0.870	0.132	0.264	0.069	0.627	0.930	0.364	0.260	0.126	0.227
tree length	b	-0.001	0.010	-0.009	0.011	0.005	0.004	-0.002	-0.020	0.016	0.008	0.003
	se	0.006	0.005	0.005	0.006	0.005	0.005	0.006	0.007	0.006	0.005	0.004
	p	0.835	0.062	0.061	0.088	0.346	0.325	0.754	0.003	0.010	0.106	0.466
tree duration	b	0.003	0.001	0.000	0.002	0.000	-0.002	0.000	0.001	-0.003	-0.003	-0.003
	se	0.003	0.003	0.003	0.003	0.003	0.003	0.003	0.003	0.003	0.003	0.002
	p	0.353	0.595	0.958	0.478	0.946	0.496	0.875	0.772	0.381	0.211	0.118
N unique speakers	b	-0.001	-0.011	0.010	-0.016	-0.002	-0.001	0.003	0.022	-0.021	-0.008	-0.002
	se	0.006	0.006	0.005	0.007	0.005	0.005	0.006	0.007	0.007	0.005	0.004
	p	0.886	0.049	0.045	0.017	0.722	0.770	0.640	0.002	0.002	0.142	0.587
% RG speakers	b	-0.002	-0.002	-0.006	-0.004	0.011	-0.006	0.019	0.003	0.000	0.008	0.001
	se	0.003	0.003	0.003	0.004	0.003	0.003	0.003	0.004	0.004	0.003	0.003
	p	0.506	0.525	0.027	0.356	0.001	0.036	0.000	0.396	0.904	0.005	0.753
% RI speakers	b	0.003	0.004	0.001	0.011	0.000	0.003	-0.006	-0.007	0.007	0.001	0.000
	se	0.003	0.003	0.003	0.004	0.003	0.003	0.004	0.004	0.004	0.003	0.003
	p	0.436	0.163	0.817	0.005	0.983	0.376	0.079	0.079	0.074	0.728	0.918
Constant	b	-0.198	-0.109	-0.076	0.238	0.087	0.144	0.044	0.319	-0.040	0.073	0.031
	se	0.003	0.003	0.003	0.003	0.003	0.003	0.003	0.003	0.003	0.003	0.002
	p	0.000	0.000	0.000	0.000	0.000	0.000	0.000	0.000	0.000	0.000	0.000

Table S11: **Robustness analysis for discussion characteristics with respect to extreme speech**. Meta-regression investigating how different properties of discussion trees affect the weighted average coefficients for Extreme speech (as shown in the third column in Tab. S13).

		Opinion	Sarcasm	Constructive	Excl.out	Incl.in/both	Anger	Fear	Disgust	Sadness	Enth./Hope	Pride/Joy
RG	b	-0.021	-0.021	0.025	0.004	0.014	0.009	0.008	0.012	-0.012	0.002	0.010
	se	0.006	0.006	0.006	0.007	0.007	0.007	0.007	0.007	0.008	0.007	0.006
	p	0.001	0.001	0.000	0.564	0.048	0.205	0.291	0.113	0.136	0.775	0.066
RI	b	-0.024	-0.028	0.028	-0.003	0.006	0.020	0.003	0.011	-0.007	0.006	0.013
	se	0.006	0.007	0.007	0.008	0.007	0.007	0.007	0.008	0.008	0.007	0.006
	p	0.000	0.000	0.000	0.741	0.416	0.007	0.707	0.136	0.382	0.350	0.028
root: journalist	b	-0.006	0.005	-0.012	-0.005	-0.005	-0.002	-0.002	-0.001	0.002	-0.004	0.004
	se	0.004	0.004	0.004	0.004	0.004	0.004	0.004	0.004	0.005	0.004	0.003
	p	0.090	0.156	0.002	0.249	0.240	0.647	0.603	0.810	0.642	0.276	0.265
root: politician	b	0.002	0.005	-0.014	0.000	0.002	-0.009	0.001	0.005	0.005	0.000	0.001
	se	0.003	0.003	0.003	0.004	0.004	0.004	0.004	0.004	0.004	0.004	0.003
	p	0.652	0.106	0.000	0.906	0.570	0.017	0.792	0.223	0.238	0.916	0.758
tree length	b	0.009	0.012	-0.008	-0.014	-0.001	0.000	0.003	-0.002	0.002	-0.009	-0.001
	se	0.006	0.006	0.006	0.007	0.007	0.007	0.007	0.007	0.008	0.007	0.005
	p	0.151	0.072	0.201	0.057	0.837	0.965	0.687	0.798	0.776	0.155	0.800
tree duration	b	-0.005	-0.005	-0.006	-0.009	-0.006	0.003	-0.003	0.002	0.005	-0.002	-0.002
	se	0.003	0.003	0.003	0.004	0.004	0.004	0.004	0.004	0.004	0.003	0.003
	p	0.102	0.151	0.063	0.017	0.111	0.471	0.417	0.586	0.201	0.571	0.592
N unique speakers	b	-0.009	-0.012	0.003	0.014	0.003	-0.005	0.001	0.001	-0.004	0.007	-0.001
	se	0.007	0.007	0.007	0.008	0.007	0.008	0.008	0.008	0.008	0.007	0.006
	p	0.170	0.084	0.617	0.080	0.632	0.521	0.914	0.881	0.612	0.351	0.891
% RG speakers	b	-0.013	-0.016	0.009	0.024	0.020	-0.009	0.031	0.007	-0.009	-0.011	0.008
	se	0.004	0.004	0.004	0.005	0.004	0.004	0.004	0.004	0.005	0.004	0.003
	p	0.001	0.000	0.017	0.000	0.000	0.049	0.000	0.138	0.056	0.005	0.020
% RI speakers	b	0.006	0.013	-0.005	-0.017	-0.003	0.007	-0.012	0.000	0.001	0.002	0.000
	se	0.004	0.004	0.004	0.005	0.004	0.004	0.004	0.004	0.005	0.004	0.003
	p	0.090	0.001	0.210	0.000	0.458	0.133	0.007	0.940	0.766	0.623	0.952
Constant	b	-0.122	-0.106	0.142	0.179	0.116	0.110	0.110	-0.005	0.011	0.056	0.076
	se	0.003	0.003	0.003	0.004	0.004	0.004	0.004	0.004	0.004	0.004	0.003
	p	0.000	0.000	0.000	0.000	0.000	0.000	0.000	0.238	0.012	0.000	0.000

Table S12: **Robustness analysis for discussion characteristics with respect to extreme speakers**. Meta-regression investigating how different properties of discussion trees affect the weighted average coefficients for Extreme speakers (as shown in the fourth column in Tab. S13).

		Opinion	Sarcasm	Constructive	Excl.out	Incl.in/both	Anger	Fear	Disgust	Sadness	Enth./Hope	Pride/Joy
RG	b	-0.017	-0.005	0.010	0.000	0.006	0.003	0.011	0.007	-0.002	0.001	0.010
	se	0.006	0.006	0.006	0.007	0.007	0.007	0.007	0.008	0.008	0.007	0.006
	p	0.007	0.466	0.108	0.994	0.382	0.685	0.122	0.338	0.791	0.926	0.108
RI	b	-0.024	-0.007	-0.001	-0.002	0.006	0.003	0.010	-0.001	-0.002	-0.004	0.005
	se	0.007	0.006	0.007	0.007	0.007	0.007	0.007	0.008	0.008	0.007	0.006
	p	0.000	0.281	0.828	0.780	0.407	0.704	0.182	0.928	0.821	0.530	0.379
root: journalist	b	0.008	0.001	-0.004	0.008	-0.004	0.003	-0.008	-0.007	0.004	-0.002	0.001
	se	0.004	0.004	0.004	0.004	0.004	0.004	0.004	0.005	0.005	0.004	0.004
	p	0.047	0.785	0.270	0.064	0.384	0.524	0.075	0.162	0.401	0.686	0.804
root: politician	b	0.002	0.002	0.000	0.008	0.008	0.001	-0.005	0.003	0.004	-0.001	-0.001
	se	0.003	0.003	0.003	0.004	0.004	0.004	0.004	0.004	0.004	0.004	0.003
	p	0.498	0.457	0.935	0.044	0.056	0.707	0.173	0.503	0.351	0.730	0.789
tree length	b	-0.005	-0.001	-0.013	-0.005	0.001	-0.004	0.006	0.004	-0.005	-0.006	0.000
	se	0.007	0.006	0.006	0.007	0.007	0.006	0.007	0.008	0.008	0.007	0.006
	p	0.449	0.863	0.045	0.435	0.939	0.475	0.399	0.606	0.562	0.363	0.942
tree duration	b	-0.003	-0.006	0.000	-0.007	-0.001	0.005	-0.004	-0.007	0.013	-0.002	0.004
	se	0.003	0.003	0.003	0.004	0.004	0.003	0.004	0.004	0.004	0.004	0.003
	p	0.429	0.089	0.929	0.060	0.732	0.182	0.317	0.092	0.002	0.641	0.243
N unique speakers	b	0.009	0.009	0.014	0.010	0.000	0.006	-0.006	-0.006	0.002	0.005	0.000
	se	0.007	0.006	0.007	0.007	0.007	0.007	0.007	0.008	0.009	0.007	0.006
	p	0.199	0.156	0.043	0.193	0.977	0.371	0.390	0.477	0.786	0.498	0.999
% RG speakers	b	-0.009	-0.020	0.019	0.015	0.008	0.004	0.013	0.005	-0.001	0.007	0.007
	se	0.004	0.004	0.004	0.005	0.004	0.004	0.004	0.005	0.005	0.004	0.004
	p	0.016	0.000	0.000	0.001	0.076	0.319	0.002	0.254	0.765	0.089	0.058
% RI speakers	b	0.006	0.010	0.009	-0.006	0.000	0.002	-0.012	0.001	0.006	0.009	0.000
	se	0.004	0.004	0.004	0.004	0.004	0.004	0.004	0.005	0.005	0.004	0.004
	p	0.134	0.008	0.027	0.181	0.953	0.565	0.006	0.913	0.209	0.037	0.893
Constant	b	-0.080	-0.078	0.035	0.054	0.037	0.016	0.062	0.021	-0.002	0.020	0.033
	se	0.003	0.003	0.003	0.004	0.004	0.004	0.004	0.004	0.004	0.004	0.003
	p	0.000	0.000	0.000	0.000	0.000	0.000	0.000	0.000	0.690	0.000	0.000

S12 Regression coefficients

S12.1 Tree-level ARDL

Table S13: **Weighted average coefficients and robust standard errors from meta-analyses of ARDL coefficients**. Coefficients were obtained on 3,569 trees with at least 50 tweets, for the relationship of different measures of quality of discourse (hate speech, toxicity, and extremity of speech and speakers) with different dimensions of discourse. Variables without prefix are the next-tweet effects, while those starting with “L1” and “L2” are lagged effects on the 2nd and 3rd next tweets, respectively.

Variable	Hate speech	Toxicity	Extreme speech	Extreme speakers
L1_self	-0.02 (0.01)	-0.02 (0.01)	-0.03 (0.01)	0.01 (0.01)
L2_self	-0.10 (0.03)	-0.10 (0.03)	-0.11 (0.03)	-0.09 (0.03)
Opinion	-0.34 (0.01)	-0.20 (0.01)	-0.12 (0.01)	-0.08 (0.01)
Sarcasm	-0.24 (0.01)	-0.11 (0.01)	-0.11 (0.01)	-0.08 (0.01)
Constructive	-0.02 (0.01)	-0.08 (0.01)	0.14 (0.01)	0.03 (0.01)
Excl.outgroup	0.17 (0.01)	0.24 (0.01)	0.18 (0.01)	0.05 (0.01)
Incl. in/both groups	0.11 (0.01)	0.09 (0.01)	0.12 (0.01)	0.04 (0.01)
Anger	0.00 (0.01)	0.15 (0.01)	0.11 (0.01)	0.02 (0.01)
Fear	0.19 (0.01)	0.04 (0.01)	0.11 (0.01)	0.06 (0.01)
Disgust	0.05 (0.01)	0.32 (0.01)	0.00 (0.01)	0.02 (0.02)
Sadness	-0.01 (0.01)	-0.04 (0.01)	0.01 (0.02)	0.00 (0.02)
Enth./Hope	0.08 (0.01)	0.07 (0.01)	0.06 (0.01)	0.02 (0.01)
Pride/Joy	0.03 (0.01)	0.03 (0.01)	0.08 (0.01)	0.03 (0.01)
L1_Opinion	-0.02 (0.02)	-0.02 (0.02)	-0.01 (0.03)	-0.01 (0.03)
L1_Sarcasm	-0.01 (0.02)	0.00 (0.02)	-0.02 (0.03)	-0.01 (0.03)
L1_Constructive	0.00 (0.02)	0.00 (0.02)	0.01 (0.03)	0.02 (0.03)
L1_Excl.outgroup	0.02 (0.03)	0.02 (0.03)	0.00 (0.03)	0.00 (0.03)
L1_Incl. in/both groups	0.01 (0.03)	0.01 (0.02)	0.01 (0.03)	-0.01 (0.03)
L1_Anger	-0.01 (0.03)	0.00 (0.02)	0.02 (0.03)	0.01 (0.03)
L1_Fear	0.02 (0.02)	0.00 (0.02)	0.01 (0.03)	0.01 (0.03)
L1_Disgust	0.00 (0.03)	0.03 (0.03)	-0.01 (0.03)	0.00 (0.03)
L1_Sadness	0.00 (0.03)	0.00 (0.02)	-0.01 (0.03)	0.01 (0.03)
L1_Enth./Hope	0.00 (0.02)	0.01 (0.02)	0.00 (0.03)	0.00 (0.03)
L1_Pride/Joy	0.00 (0.02)	-0.01 (0.02)	0.01 (0.03)	0.02 (0.03)
L2_Opinion	0.00 (0.04)	-0.04 (0.04)	-0.01 (0.04)	-0.02 (0.05)
L2_Sarcasm	-0.03 (0.04)	-0.02 (0.04)	-0.01 (0.05)	0.00 (0.05)
L2_Constructive	0.00 (0.04)	0.00 (0.03)	0.00 (0.04)	0.01 (0.05)
L2_Excl.outgroup	0.04 (0.05)	0.05 (0.05)	0.04 (0.05)	0.01 (0.05)
L2_Incl. in/both groups	0.01 (0.05)	0.01 (0.04)	0.00 (0.05)	0.01 (0.05)
L2_Anger	-0.02 (0.05)	0.03 (0.04)	0.01 (0.05)	0.00 (0.05)
L2_Fear	0.02 (0.04)	0.02 (0.04)	0.03 (0.05)	0.02 (0.05)
L2_Disgust	0.01 (0.05)	0.04 (0.04)	0.00 (0.05)	0.01 (0.05)
L2_Sadness	0.01 (0.05)	0.00 (0.04)	0.01 (0.05)	0.00 (0.06)
L2_Enth./Hope	0.00 (0.04)	0.02 (0.04)	0.02 (0.05)	-0.01 (0.05)
L2_Pride/Joy	0.00 (0.04)	0.02 (0.04)	0.01 (0.04)	0.03 (0.05)
Tweet order	0.00 (0.00)	0.00 (0.00)	0.00 (0.00)	0.00 (0.00)
Constant	0.01 (0.02)	-0.02 (0.01)	0.00 (0.02)	-0.03 (0.02)

S12.2 Day-level ARDL

Table S14: **Day-to-day analysis of the relationship of different measures of quality of discourse with different dimensions of discourse**. Quality measures are hate speech, toxicity, and extremity of speech and speakers. Variables without prefix are the same-day effects, while those starting with “L1” and “L2” are lagged effects on the 1st and 2nd following day, respectively. Robust standard errors are in brackets. Variables starting with “RG” are interactions with the presence of Reconquista Germanica, and those starting with “RI” are interactions with the presence of both extreme groups (RG and Reconquista Internet) in the public sphere. Results are from ARDL models estimated on 1,461 consecutive days from January 1, 2015 to December 31, 2018.

Variable	Hate speech	Toxicity	Extreme speech	Extreme speakers
L1_self	0.29 (0.03)	0.20 (0.03)	0.19 (0.03)	0.18 (0.03)
L2_self	0.11 (0.02)	0.15 (0.03)	0.13 (0.02)	0.21 (0.03)
Opinion	-0.24 (0.03)	-0.19 (0.03)	-0.15 (0.04)	-0.17 (0.05)
Sarcasm	-0.03 (0.04)	0.07 (0.05)	-0.12 (0.05)	-0.09 (0.07)
Constructive	0.02 (0.03)	-0.24 (0.04)	0.25 (0.05)	-0.16 (0.06)
Excl.outgroup	0.52 (0.04)	0.38 (0.04)	0.37 (0.05)	0.42 (0.06)
Incl. in/both groups	0.32 (0.04)	0.10 (0.05)	0.20 (0.05)	0.20 (0.07)
Anger	-0.11 (0.04)	0.19 (0.05)	0.05 (0.06)	-0.15 (0.07)
Fear	0.21 (0.03)	0.16 (0.03)	0.20 (0.04)	0.19 (0.06)
Disgust	0.12 (0.04)	0.40 (0.05)	0.00 (0.06)	-0.07 (0.07)
Sadness	-0.11 (0.04)	-0.07 (0.04)	-0.02 (0.05)	-0.01 (0.07)
Enth./Hope	-0.04 (0.03)	0.03 (0.03)	0.05 (0.04)	-0.03 (0.05)
Pride/Joy	0.02 (0.02)	0.05 (0.03)	0.04 (0.03)	-0.01 (0.04)
L1_Opinion	0.08 (0.03)	0.08 (0.02)	0.05 (0.03)	
L1_Sadness	0.05 (0.03)		-0.05 (0.02)	
L1_Constructive		0.00 (0.03)	-0.02 (0.04)	0.02 (0.05)
L1_Excl.outgroup	-0.12 (0.03)	-0.03 (0.03)	-0.10 (0.03)	
L1_Incl. in/both groups	-0.04 (0.03)		-0.05 (0.04)	
L1_Anger		-0.06 (0.03)		-0.07 (0.04)
L1_Fear	-0.08 (0.02)	0.03 (0.02)		
L1_Disgust	-0.02 (0.03)	-0.10 (0.03)		-0.04 (0.03)
L1_Sarcasm				0.07 (0.04)
L1_Enth./Hope		-0.06 (0.02)	-0.03 (0.03)	-0.04 (0.03)
L1_Pride/Joy	-0.02 (0.01)			-0.06 (0.03)
L2_Opinion			0.05 (0.03)	
L2_Constructive		0.11 (0.03)	-0.06 (0.03)	0.07 (0.04)
L2_Sarcasm				0.08 (0.04)
L2_Excl.outgroup		-0.04 (0.02)		
L2_Anger				0.09 (0.03)
L2_Disgust	-0.04 (0.02)	-0.10 (0.03)	-0.03 (0.02)	-0.06 (0.03)
L2_Sadness			-0.04 (0.02)	
L2_Enth./Hope		-0.03 (0.02)		
RG	0.11 (0.06)	0.00 (0.07)	-0.09 (0.08)	-0.12 (0.10)
RG_Opinion	-0.19 (0.06)	-0.21 (0.06)	-0.22 (0.07)	-0.11 (0.09)
RG_Sarcasm	-0.03 (0.07)	-0.16 (0.08)	-0.13 (0.09)	-0.31 (0.12)
RG_Constructive	0.03 (0.07)	0.00 (0.07)	0.14 (0.08)	0.07 (0.11)
RG_Excl.outgroup	-0.01 (0.08)	-0.06 (0.08)	0.02 (0.09)	-0.19 (0.11)
RG_Incl. in/both groups	0.13 (0.06)	0.21 (0.06)	0.15 (0.08)	0.01 (0.09)
RG_Anger	0.00 (0.08)	0.12 (0.08)	0.10 (0.09)	-0.02 (0.12)
RG_Fear	-0.01 (0.05)	-0.08 (0.05)	0.08 (0.07)	-0.03 (0.08)
RG_Disgust	0.02 (0.07)	0.02 (0.07)	-0.02 (0.09)	0.20 (0.11)
RG_Sadness	0.04 (0.07)	0.09 (0.07)	0.10 (0.09)	0.09 (0.11)
RG_Enth./Hope	0.04 (0.05)	0.04 (0.05)	0.05 (0.06)	0.07 (0.07)
RG_Pride/Joy	-0.01 (0.04)	0.05 (0.04)	0.09 (0.05)	0.05 (0.06)
RI	0.36 (0.16)	0.28 (0.14)	0.28 (0.17)	0.53 (0.20)
RI_Opinion	-0.18 (0.11)	-0.22 (0.09)	0.14 (0.11)	-0.10 (0.14)
RI_Sarcasm	0.07 (0.13)	-0.07 (0.11)	0.11 (0.16)	-0.02 (0.17)
RI_Constructive	0.01 (0.12)	-0.08 (0.11)	0.29 (0.13)	0.38 (0.16)
RI_Excl.outgroup	0.31 (0.15)	-0.12 (0.13)	0.36 (0.18)	0.06 (0.18)
RI_Incl. in/both groups	0.19 (0.09)	0.17 (0.08)	0.16 (0.10)	0.13 (0.12)
RI_Anger	-0.44 (0.14)	0.00 (0.13)	-0.17 (0.16)	0.02 (0.17)
RI_Fear	0.15 (0.09)	-0.05 (0.08)	0.15 (0.11)	0.04 (0.11)
RI_Disgust	0.06 (0.11)	0.12 (0.09)	-0.04 (0.12)	0.20 (0.14)
RI_Sadness	-0.02 (0.13)	0.04 (0.11)	0.02 (0.13)	-0.25 (0.17)
RI_Enth./Hope	0.01 (0.08)	-0.01 (0.07)	-0.09 (0.09)	-0.04 (0.10)
RI_Pride/Joy	-0.14 (0.07)	0.05 (0.06)	-0.03 (0.06)	0.06 (0.08)
Day order	0.00 (0.00)	0.00 (0.00)	0.00 (0.00)	0.00 (0.00)
Constant	0.03 (0.13)	0.62 (0.16)	-0.43 (0.19)	-0.38 (0.26)

S13 REFORMS checklist

Below we complete the REFORMS checklist [41] which provides consensus-based recommendations for reporting machine-learning-based science.

Module 1: Study goals

1a. Population or distribution about which the scientific claim is made.

Users partaking in political discussions on Twitter in Germany between January 2015 and Dezember 2018 (main text pp. 2-3).

1b. Motivation for choosing this population or distribution (1a.).

Our sample is especially suitable for the study of hate and counter speech, since during the period of study dedicated hate and counter speech groups were active on Twitter (Reconquista Germanica & Reconquista Internet). Their activity allows for the study of the effectiveness of different counter speech strategies in an ecologically valid environment. (main text pp. 2-3)

1c. Motivation for the use of ML methods in the study.

To date, counter speech has only been studied in the laboratory or through the analysis of a small number of selected online conversations. Machine learning enabled us to classify hate speech, different counter speech strategies, and socio-psychological goals at scale. Our study thus provides the first ever large-scale assessment of hate and counter speech in online political discussions with more than 130,000 conversations and over 1 million tweets (main text p. 2).

Module 2: Computational reproducibility

2a. Dataset used for training and evaluating the model along with link or DOI to uniquely identify the dataset.

We trained our models on a sample of n = 14,692 expert annotated tweets balanced over year and extremity of speakers. Another sample of n = 1,000 served as a held-out test set to evaluate the final classifiers, which we never used during the training process. See Section 4.2 in the main and Sections S2 and S3 in the SI.
See also our data availability statement (main text p. 49): "Following the Twitter terms of service, we are not allowed to publish the texts of the tweets contained in our corpus. We do, however, publish all inferred information necessary to reproduce the ARDL analysis presented in the paper. This data is available under accession code 10.17605/OSF.IO/X4WE6. The data can be provided by the authors pending scientific review and a completed material transfer agreement. Requests for the data should be submitted to alina.herderich@uni-graz.at or jana.lasser@uni-graz.at."

2b. Code used to train and evaluate the model and produce the results reported in the paper along with link or DOI to uniquely identify the version of the code used.

The code is published at https://github.com/JanaLasser/counterspeech-strategies. We will create release of the code with a citeable DOI upon completion of the revision process (see also code availability statement, main text p. 49).

2c. Description of the computing infrastructure used.

We used a single NVIDIA Quadro RTX 8000 GPU with 48 GB GDDR6 memory for training, which included masked language modeling and fine-tuning, and inference. The masked language modeling task took 142 hours to complete. Fine-tuning the pre-trained model for the different classifiers usually reached the early stopping criterion after 2-3 epochs and took 10-15 minutes. Since several models were trained for different classifiers, different data splits and different stages of data augmentation, we trained around 50 models, amounting to about half a day of training time. (SI, p. 32). To perform the fine-tuning, we used the 'transformers' library for Python (version 4.11.3), see also SI, p. 34.

2d. README file which contains instructions for generating the results using the provided dataset and code.

The README file of the repository (see https://github.com/JanaLasser/counterspeech-strategies) is currently a work in progress. As the paper undergoes peer review, we will update it to provide a complete set of instructions to generate the results using the provided dataset and code.

2e. Reproduction script to produce all results reported in the paper.

Since we cannot publish the raw data used to train the machine learning models, we also cannot provide a functional script to reproduce the training of the models. Pending review, we will however publish the trained models and scripts necessary for inference and reproduction of the results given the trained models.

Module 3: Data quality

3a. Source(s) of data, separately for the training and evaluation datasets (if applicable), along with the time when the dataset(s) are collected, the source and process of ground-truth annotations, and other data documentation.

The data set is based on a previous study [3]. The data set contains conversations on Twitter under posts of major German news organizations, journalists, bloggers and politicians (for a list of all 22 accounts see Ref. [3]). The data was collected through a custom-made web scraper tool between 2015 and 2018 and stopped after, because of a change in Twitter's html files that made scraping much harder. All conversations below these accounts were collected in the

aforementioned time period. Training and evaluation samples for human annotation were drawn separately from this data set. Data was annotated by four experts (psychologists or master psychology students, see Section S3.1 in the SI) on the dimensions argumentation strategy, in- and outgroup content, socio-psychological goal, and hate speech (see Section S2 in the SI). We labeled 15,692 tweets and the labeling took around 9 months to complete.

3b. Distribution or set from which the dataset is sampled (i.e., the sampling frame).

We collected conversation trees stemming from the posts of 22 major German news actors on Twitter between 2015 and 2018, which included 1,150,469 tweets from 130,548 different users. Samples for human annotation were drawn randomly from this set, but balanced over year and extremity of speech, and with a tendency to oversample minority classes to create a balanced training data set (see Section S3.3 in the SI).

3c. Justification for why the dataset is useful for the modeling task at hand.

Two dedicated hate and counter speech groups were active during that time on German Twitter (Reconquista Germanica, Reconquista Internet). We are therefore sure to observe our main concepts of interest: hate and counter speech. The time between 2015 and 2018 sparked a lot of political discussions in Germany because of political events like the "migrant crisis". We observe political discussions in the wild rather than in the laboratory, painting a more realistic picture about naturally occurring citizen organized counter speech (see main text pp. 2-3).

3d. The definition of the outcome variable of the model along with descriptive statistics, if applicable.

We trained three separate classification models for outcome variables argumentation strategy, socio-psychological goal, and hate speech. We further use existing, previously validated models out of the box for emotions, toxicity, and extremity of speech. In our statistical models (ARDL & matching), dependent variables are hate speech, toxicity, and extremity of speech; and independent variables are argumentation strategy, socio-psychological goal, and emotions. Time series for all dependent and independent variables are displayed in Figure 1 (main text p. 24). The number of training samples going into each model of argumentation strategy, socio-psychological goal, and hate speech is displayed in Table S4 (SI p. 36). Correlations between independent and dependent variables are displayed in Table S7 (SI p. 53). The outcome variable is also known as the dependent variable, the target variable, the output variable or the predicted variable.

3e. Number of samples in the dataset.

Our data set contains 1,150,469 tweets in 130,127 conversations originating from 130,548 users (main text p. 11). Human annotators labeled 15,692 tweets for the training and evaluation of our custom-made classifiers (main text p. 12). The number of training samples going into each model of argumentation strategy, socio-psychological goal, and hate speech based on a staged training procedure is displayed in Table S4 (SI p. 36).

3f. Percentage of missing data, split by class for a categorical outcome variable.

"This data set consists of 130,394 trees containing 1,167,853 tweets from 134,092 unique users. [...] After excluding tweets for which we were not able to calculate toxicity scores, we ended up with 130,127 trees containing 1,150,469 tweets from 130,548 unique users." (main text p. 11) Author's note: Being unable to compute toxicity scores could be traced back to tweets only containing a picture or link or similar. See also this checklist 4b.

3g. Justification for why the distribution or set from which the dataset is drawn (3b.) is representative of the one about which the scientific claim is being made (1a.).

We do not claim that our sample is representative of the German population, nor the average internet user, like no sample except for representative surveys is. However, we note that social media has become a central part of many people's lives blending the offline and online world in nearly all areas of life including politics. We further note that, to the best of our knowledge, our sample is the largest ever observation of naturally occurring political discussions, hate and counter speech up until today.

Module 4: Data preprocessing

4a. Identification of whether any samples are excluded with a rationale for why they are excluded.

See 3f.

4b. How impossible or corrupt samples are dealt with.

For each round of human annotations author AH checked the completeness and category assignments of data points and followed up with the annotators to clarify potential errors. Hence, training and evaluation data sets are entirely clean.

4c. All transformations of the dataset from its raw form (3a.) to the form used in the model, for instance, treatment of missing data and normalization.

We did not perform any preprocessing of the documents contained in the corpus. We did, however, implement a data augmentation strategy: We first

finetuned a "twitter-xlm-roberta-base" model with a masked language modeling task on our data set (SI p. 32). Based on the human annotated training dataset, we implemented a staged training procedure to train each of our three classification models (argumentation strategy, socio-psychological goal, hate speech): For the first round of training, we identified "confident samples" where human annotators agreed. For the second and third round of training, we selected tweets where one human annotator and the preliminary version of the classifier (from steps one or two) agreed (SI pp. 32-35). In each training round, we augmented our training data set with back translation (SI p. 33). The number of training samples going into each model for each round of training is displayed in Table S4 (SI p. 36).

Module 5: Modeling

5a. Detailed descriptions of all models trained, including:

- All features used in the model (including any feature selection).
- Types of models implemented (e.g., Random Forests, Neural Networks).
- Loss function used.

We used a pre-trained language model, namely "twitter-xml-roberta-base". The model is publicly available: https://huggingface.co/cardiffnlp/twitter-xlm-roberta-base (main text p.12 and SI p. 32). We used the default tokenizer of the same model with the following settings to embed the texts and generate the features: `encodings = tokenizer(text, truncation=True, padding=True, max_length=180)`. All hyperparameter settings are specified on p. 34 of the SI including the loss function (macro-F1 score).

5b. Justification for the choice of model types implemented.

Twitter-xlm-roberta-base" is a multilingual RoBERTa based transformer model, which was a state-of-the-art natural language model at the time of study. It was pre-trained on Twitter data making it especially suitable for our data set. We compared our model of choice against a German BERT at an early stage of the process, which resulted in similar classification performance, but with greater variability, which is why we chose "twitter-xlm-roberta-base" as our base model moving forward.

5c. Method for evaluating the model(s) reported in the paper, including details of train-test splits or cross-validation folds.

We fine-tuned our models with five fold cross validation and a staged training procedure (see 4c.). We determined the best model of those five for each of argumentation strategy, socio-psychological goal, and hate speech as our final classifiers. We evaluated the three final classifiers against a held-out human annotated test set of 1,000 tweets that has never been used for training. More specifically, we evaluated our model against all cases in the test set where 3 out of 4 human annotators agreed (677, 127, and 900 tweets, respectively; main

text pp. 12-13). AUC scores, ROC curves, and F1 scores are reported in the SI in Table S5, Figure S3, and Figure S4.

5d. Method for selecting the model(s) reported in the paper.

See 5b., 5c., and 4c.

5e. For the model(s) reported in the paper, specify details about the hyperparameter tuning:

- Range of hyper-parameters used and a justification for why this range is reasonable.
- Method to select the best hyper-parameter configuration.
- Specification of all hyper-parameters used to generate results reported in the paper.

We performed masked language modeling over 100 epochs, using a randomly selected sample of 20% of the corpus as validation set. MLM was performed with the following set of parameters: a learning rate of $2 \cdot 10^{-5}$, a weight decay of 0.01, 8 gradient accumulation steps, a batch size of 64, and a masking probability of 15%. Training performance was evaluated every epoch. The MLM task took 142 hours to complete on a single NVIDIA Quadro RTX 8000 GPU with 48 GB GDDR6 memory and reduced model perplexity on the validation set to 6.05 (SI p. 32).
To find optimal hyperparameters for model fine-tuning, we performed a random search for the model fine-tuned on STRATEGY and used the thus found hyperparameters for the HATE, GROUP and GOAL models as well. We did not perform separate hyperparameter searches for each model due to the computational cost. The random search was performed in the following parameter space: learning rate: $[1 \cdot 10^{-5}, 5 \cdot 10^{-}5, 1 \cdot 10^{-}4]$, weight decay: [0.001, 0.0025, 0.005], label smoothing factor: [0.1, 0.2, 0.3], training batch size: [32, 64, 128, 256, 512].
If not noted otherwise, we used the following set of model parameters found via the hyperparameter search for each supervised prediction task described in the remainder of the section: a learning rate of $5 \cdot 10^{-5}$, weight decay of 0.0025, a label smoothing factor of 0.2 and a training batch size of 256. Evaluation was performed every 5 training steps with the macro-F1 score as the evaluation metric. Fine-tuning was done for a maximum of 10 epochs with early stopping after 5 evaluation steps with consecutively worse performance and 100 warmup steps. The maximum text length was set to 180 tokens.” (SI p.34)

5f. Justification that model comparisons are against appropriate baselines.

We compare against a frequency-adjusted random guessing baseline. While this is a relatively “stupid” baseline, it still provides an easy way to assess how much better the model performs than chance, which is our intention.

Module 6: Data leakage

6a. Justification that pre-processing (Section 4) and modeling (Section 5) steps only use information from the training dataset (and not the test dataset).

"Note that the final classifiers were validated against the held-out test set labeled by human annotators that we never used for fine-tuning [...]" (SI p. 34). See details on the test set in SI Section S3.2.

6b. Methods to address dependencies or duplicates between the training and test datasets (e.g. different samples from the same patients are kept in the same dataset partition).

"To create a held-out test set for the final validation of the classifiers [...], we drew another random sample of n=1,000 tweets balanced across time and extremity of speakers." (SI, p. 9)

6c. Justification that each feature or input used in the model is legitimate for the task at hand and does not lead to leakage.

The only input to our classifiers are the embeddings of the tweet texts. Training data is sampled across all years and extremity of speech to provide the most accurate estimate for argumentation strategy, socio-psychological goal, and hate speech for the complete data set.

Module 7: Metrics and uncertainty

7a. All metrics used to assess and compare model performance (e.g., accuracy, AUROC etc.). Justify that the metric used to select the final model is suitable for the task.

"We assess the receiver-operator characteristic of our classifiers and calculate the area under the curve (AUC) – a common metric to assess the performance of machine learning classifiers. ROC curves are calculated by comparing predicted class probabilities from each model (trained on different data splits) to human labels in the held-out test set. AUC values between 0.8 and 0.9 are typically considered as 'excellent discrimination' while values between 0.7 and 0.8 are still considered as 'acceptable discrimination'. AUC values for our classifiers range from 0.73 ± 0.01[...] to 0.94 ± 0.01 [...]. The AUC values for every class are reported in Table S5 in the SI while the ROC-curves for every class are reported in Fig. S3 in the SI.
We also transformed the predicted class probabilities into class labels by assigning each example to the class with the maximum probability. We report the average classifier precision, recall and F1-score compared to a frequency-based random guessing benchmark for every class in Fig. S4 in the SI." (p. 12-13)

7b. Uncertainty estimates (e.g., confidence intervals, standard deviations), and details of how these are calculated.

We report standard deviations for ROC and AUC (SI pp. 37-28) and 95% confidence intervals for F1 scores (SI p. 39). These uncertainty estimates were calculated by training five versions of each classifier on different training/evaluation splits and then assessing performance on the test set.

7c. Justification for the choice of statistical tests (if used) and a check for the assumptions of the statistical test.

No statistical testing was performed for comparing model performance.

Module 8: Generalizability and limitations

8a. Evidence of external validity.

"Studies using human coding of subsets of discourse and experiments have produced important results, showing that following the norms of rationality (providing reasons and evidence), constructiveness (solution-oriented discourse), and politeness, appealing to moral principles and encouraging empathy for the victims can lead to a better deliberative quality and less hate in subsequent discourse. However, past studies were limited to relatively small snapshots of online discourse at a single point in time. In addition, controlled experiments on hateful behavior are nearly infeasible while preserving participant safety and ensuring informed consent. To understand the real-world interplay of hate and counter speech, we need to measure different dimensions of discourse in large textual corpora over longer periods of time." (p. 2)
"We show that our results are robust across different measures of discourse quality – hate speech, toxicity, extremity of speech and speakers – as well as on micro, meso, and macro levels of analysis." (p. 9)
The study variables were developed with a grounded theory approach to best reflect naturally occurring counter speech (SI Section S2).

8b. Contexts in which the authors do not expect the study's findings to hold.

"Our results are limited to the dimensions of discourse we investigated and are not informative about other kinds of misuse of online commons beyond hatefulness and extremism, such as various forms of misinformation and fraud. That should be a topic of further research. Furthermore, we could not make reliable conclusions about some counter speech strategies that have been identified in experimental studies, such as empathy for the victims of hate speech and related moral appeals to treat the outgroup well, because we found too few examples of such strategies in our corpus. It is possible that such an empathic emotional tone would also have promoted a more civil discourse. This raises the question of whether it is more productive to teach people new, potentially more effective strategies, or encourage strategies that people are already using even though they might be somewhat less effective." (main text pp. 9-10)

S14 Material Transfer Agreement

Collective moderation of hate, toxicity, and extremity in online discussions
Material Transfer Agreement

Following the Twitter terms of service, we are not allowed to publish the texts of the tweets contained in our corpus. We do, however, publish all inferred information necessary to reproduce the ARDL analysis presented in the paper. If this is enough for your purpose, please download the data under accession code **10.17605/OSF.IO/X4WE6**. Original tweet texts can be shared upon request by signing the following agreement.

Recipient eligibility
Recipients must be graduate students or faculty of a non-profit, degree-granting, or academic institution.

Please provide the following data.
First name: ____
Last name: ____
Email: ____
Research institution: ____
Academic profile: ____

In a few sentences, please describe how you will use this dataset in your research.

Terms of Use
(1) You agree to only use this data for legitimate academic and/or scientific research, meaning no analyses, reviews, or derivative works of this dataset may be used for commercial or for-profit purposes in any way;
(2) You agree not to re-publish any new versions of this dataset, whether original or derivative (i.e., modified or updated in some way);
(3) You agree not to attempt to personally identify users, whose Tweets are contained in the dataset and were public at the time of data acquisition, beyond the information that is provided in the dataset itself.

By signing, I agree to the terms of use.

____
Date, Signature

***

To request the data, please send the complete Data Transfer Agreement via email to jana.lasser@uni-graz.at or alina.herderich@tugraz.at. If you have questions or concerns please feel free to contact us.

References

[1] [software] Jigsaw: Perspective API. https://www.perspectiveapi.com. Accessed: 2023-02-06

[2] Habermas, J.: The Theory of Communicative Action: Reason and the Rationalization of Society, Volume 1. Beacon Press, ??? (1984)

[3] [dataset] Garland, J., Ghazi-Zahedi, K., Young, J.-G., Hébert-Dufresne, L., Galesic, M.: Impact and dynamics of hate and counter speech online. EPJ Data Science **11**(1), 3 (2022). https://doi.org/10.1140/epjds/s13688-021-00314-6

[4] [software] Garland, J., Ghazi-Zahedi, K., Young, J.-G., Hébert-Dufresne, L., Galesic, M.: Countering hate on social media: Large scale classification of hate and counter speech. In: Proceedings of the Fourth Workshop on Online Abuse And Harms, pp. 102–112 (2020). https://doi.org/10.18653/v1/2020.alw-1.1

[5] Boyd, R.L., Schwartz, H.A.: Natural language analysis and the psychology of verbal behavior: The past, present, and future states of the field. Journal of Language and Social Psychology **40**(1), 21–41 (2021) https://arxiv.org/abs/https://doi.org/10.1177/0261927X20967028. https://doi.org/10.1177/0261927X20967028. PMID: 34413563

[6] Gentzkow, M., Shapiro, J.M., Taddy, M.: Measuring group differences in high-dimensional choices: Method and application to congressional speech. Econometrica **87**(4), 1307–1340 (2019) https://arxiv.org/abs/https://onlinelibrary.wiley.com/doi/pdf/10.3982/ECTA16566. https://doi.org/10.3982/ECTA16566

[7] Le, Q., Mikolov, T.: Distributed representations of sentences and documents. In: Proceedings of the 31st International Conference on International Conference on Machine Learning, vol. 32, pp. 1188–1196 (2014)

[8] Glaser, B.G., Strauss, A.: The Discovery of Grounded Theory: Strategies for Qualitative Research. Aldine Publishing Co., London (1967)

[9] Friess, D., Ziegele, M., Heinbach, D.: Collective civic moderation for deliberation? Exploring the links between citizens' organized engagement in comment sections and the deliberative quality of online discussions. Political Communication **38**(5), 624–646 (2021). https://doi.org/10.1080/10584609.2020.1830322

[10] Benesch, S., Ruths, D., Dillon, K., Saleem, H., Wright, L.: Considerations for successful counterspeech. Technical report, Dangerous Speech Project; University of Connecticut (2016).

https://dangerousspeech.org/wp-content/uploads/2016/10/Considerations-for-Successful-Counterspeech.pdf

[11] Cheng, J., Danescu-Niculescu-Mizil, C., Leskovec, J.: Antisocial behavior in online discussion communities. In: Proceedings of the International AAAI Conference on Web and Social Media, vol. 9, pp. 61–70 (2015). https://doi.org/10.1609/icwsm.v9i1.14583

[12] Wulczyn, E., Thain, N., Dixon, L.: Ex machina: Personal attacks seen at scale. In: Proceedings of the 26th International Conference on World Wide Web, pp. 1391–1399 (2017). https://doi.org/10.1145/3038912.3052591

[13] Krippendorff, K.: Estimating the reliability, systematic error and random error of interval data. Educational and Psychological Measurement **30**(1), 61–70 (1970). https://doi.org/10.1177/001316447003000105

[14] Krippendorff, K.: Content Analysis: An Introduction to Its Methodology. Sage, Thousand Oaks, California (2004)

[15] Pavlopoulos, J., Sorensen, J., Dixon, L., Thain, N., Androutsopoulos, I.: Toxicity detection: Does context really matter? In: Proceedings of the 58th Annual Meeting of the Association for Computational Linguistics, pp. 4296–4305. Association for Computational Linguistics, online (2020). https://aclanthology.org/2020.acl-main.396.pdf

[16] Ross, B., Rist, M., Carbonell, G., Cabrera, B., Kurowsky, N., Wojatzki, M.: Measuring the reliability of hate speech annotations: The case of the european refugee crisis. arXiv (2017). https://doi.org/10.48550/arXiv.1701.08118

[17] [software] Barbieri, F., Anke, L.E., Camacho-Collados, J.: XLM-T: A multilingual language model toolkit for Twitter. arXiv (2021). https://doi.org/10.48550/arXiv.2104.12250

[18] Gururangan, S., Marasović, A., Swayamdipta, S., Lo, K., Beltagy, I., Downey, D., Smith, N.A.: Don't stop pretraining: Adapt language models to domains and tasks. In: Proceedings of the 58th Annual Meeting of the Association for Computational Linguistics. Association for Computational Linguistics, ??? (2020). https://doi.org/10.18653/v1/2020.acl-main.74

[19] Shorten, C., Khoshgoftaar, T.M., Furht, B.: Text data augmentation for deep learning. Journal of Big Data **8**(1) (2021). https://doi.org/10.1186/s40537-021-00492-0

[20] Sennrich, R., Haddow, B., Birch, A.: Improving neural machine translation models with monolingual data. In: Proceedings of the 54th Annual

Meeting of the Association for Computational Linguistics, pp. 86–96 (2016). https://doi.org/10.18653/v1/P16-100

[21] Junczys-Dowmunt, M., Grundkiewicz, R., Dwojak, T., Hoang, H., Heafield, K., Neckermann, T., Seide, F., Germann, U., Fikri Aji, A., Bogoychev, N., Martins, A.F.T., Birch, A.: Marian: Fast neural machine translation in C++. In: Proceedings of ACL 2018, System Demonstrations, pp. 116–121. Association for Computational Linguistics, ??? (2018). https://doi.org/10.18653/v1/P18-402

[22] Wolf, T., Debut, L., Sanh, V., Chaumond, J., Delangue, C., Moi, A., Cistac, P., Rault, T., Louf, R., Funtowicz, M., *et al.*: Transformers: State-of-the-art natural language processing. In: Proceedings of the 2020 Conference on Empirical Methods in Natural Language Processing: System Demonstrations, pp. 38–45. Association for Computational Linguistics, ??? (2019). https://doi.org/10.18653/v1/2020.emnlp-demos.

[23] Pedregosa, F., Varoquaux, G., Gramfort, A., Michel, V., Thirion, B., Grisel, O., Blondel, M., Prettenhofer, P., Weiss, R., Dubourg, V., Vanderplas, J., Passos, A., Cournapeau, D., Brucher, M., Perrot, M., Duchesnay, E.: Scikit-learn: Machine learning in Python. Journal of Machine Learning Research **12**, 2825–2830 (2011). https://doi.org/10.48550/arXiv.1201.0490

[24] Mosqueira-Rey, E., Hernández-Pereira, E., Alonso-Ríos, D., Bobes-Bascarán, J., Fernández-Leal, Á.: Human-in-the-loop machine learning: A state of the art. Artificial Intelligence Review (2022). https://doi.org/10.1007/s10462-022-10246-w

[25] [software] Widmann, T., Wich, M.: Creating and comparing dictionary, word embedding, and transformer-based models to measure discrete emotions in German political text. Political Analysis, 1–16 (2022). https://doi.org/10.1017/pan.2022.15

[26] Marcus, G.E., Neuman, W.R., MacKuen, M.B.: Measuring emotional response: Comparing alternative approaches to measurement. Political Science Research and Methods **5**(4), 733–754 (2017). https://doi.org/10.1017/psrm.2015.65

[27] Sullivan, G.B.: Collective pride, happiness, and celebratory emotions, pp. 266–280. Oxford University Press, London (2014). https://doi.org/10.1093/acprof:oso/9780199659180.003.0018

[28] Ho, D.E., Imai, K., King, G., Stuart, E.A.: Matching as Nonparametric Preprocessing for Reducing Model Dependence in Parametric Causal Inference. Political Analysis **15**, 199–236 (2007). https://doi.org/10.1093/pan/mpl013

[29] Ho, D., Imai, K., King, G., Stuart, E.A.: MatchIt: Nonparametric Preprocessing for Parametric Causal Inference. Journal of Statistical Software **42**, 1–28 (2011). https://doi.org/10.18637/jss.v042.i08

[30] Nguyen, T.-L., Collins, G.S., Spence, J., Daurès, J.-P., Devereaux, P., Landais, P., Le Manach, Y.: Double-adjustment in propensity score matching analysis: Choosing a threshold for considering residual imbalance. BMC Medical Research Methodology **17**, 1–8 (2017). https://doi.org/10.1186/s12874-017-0338-0

[31] Arel-Bundock, V., Diniz, M.A., Greifer, N., Bacher, E.: Marginaleffects: Predictions, Comparisons, Slopes, Marginal Means, and Hypothesis Tests (2023)

[32] Card, D., Chang, S., Becker, C., Mendelsohn, J., Voigt, R., Boustan, L., Abramitzky, R., Jurafsky, D.: Computational analysis of 140 years of US political speeches reveals more positive but increasingly polarized framing of immigration. Proceedings of the National Academy of Sciences **119**(31), 2120510119 (2022). https://doi.org/10.1073/pnas.2120510119

[33] [software] Kripfganz, S., Schneider, D.C., *et al.*: ARDL: Estimating autoregressive distributed lag and equilibrium correction models. In: Proceedings of the 2018 London Stata Conference, p. 59 (2018)

[34] Kripfganz, S., Schneider, D.C.: ardl: Estimating autoregressive distributed lag and equilibrium correction models. The Stata Journal **23**(4), 983–1019 (2023)

[35] Dickey, D.A., Fuller, W.A.: Distribution of the estimators for autoregressive time series with a unit root. Journal of the American Statistical Association **74**(366a), 427–431 (1979). https://doi.org/10.2307/2286348

[36] Pesaran, M.H., Shin, Y., Smith, R.J.: Bounds testing approaches to the analysis of level relationships. Journal of Applied Econometrics **16**(3), 289–326 (2001). https://doi.org/10.1002/jae.616

[37] White, H.: A heteroskedasticity-consistent covariance matrix estimator and a direct test for heteroskedasticity. Econometrica: Journal of the Econometric Society, 817–838 (1980). https://doi.org/10.2307/1912934

[38] Godfrey, L.G.: Misspecification tests and their uses in econometrics. Journal of Statistical Planning and Inference **49**(2), 241–260 (1996). https://doi.org/10.1016/0378-3758(95)00039-9

[39] Brown, R.L., Durbin, J., Evans, J.M.: Techniques for testing the constancy of regression relationships over time. Journal of the Royal Statistical Society: Series B (Methodological) **37**(2), 149–163 (1975)

[40] Natsiopoulos, K.: ARDL in R. (2023). R package version 0.2.4. https://cran.r-project.org/web/packages/ARDL/ARDL.pdf

[41] Kapoor, S., Cantrell, E.M., Peng, K., Pham, T.H., Bail, C.A., Gundersen, O.E., Hofman, J.M., Hullman, J., Lones, M.A., Malik, M.M., *et al.*: REFORMS: Consensus-based recommendations for machine-learning-based science. Science Advances **10**(18), 3452 (2024). https://doi.org/10.1126/sciadv.adk3452
